# Dietary serine-microbiota interaction enhances chemotherapeutic toxicity without altering drug conversion

**DOI:** 10.1038/s41467-020-16220-w

**Published:** 2020-05-22

**Authors:** Wenfan Ke, James A. Saba, Cong-Hui Yao, Michael A. Hilzendeger, Anna Drangowska-Way, Chintan Joshi, Vinod K. Mony, Shawna B. Benjamin, Sisi Zhang, Jason Locasale, Gary J. Patti, Nathan Lewis, Eyleen J. O’Rourke

**Affiliations:** 1 Department of Biology, College of Arts and Sciences, University of Virginia, Charlottesville, VA USA; 2 Department of Chemistry, Washington University, St. Louis, MO USA; 3 Department of Pediatrics, University of California, San Diego, La Jolla, CA USA; 4 Department of Bioengineering, University of California, San Diego, La Jolla, CA USA; 5 Department of Pharmacology and Cancer Biology, Duke University, Durham, NC USA; 6 Department of Cell Biology, School of Medicine, University of Virginia, Charlottesville, VA USA

**Keywords:** Pharmacodynamics, Bacterial physiology, Microbiome, Symbiosis

## Abstract

The gut microbiota metabolizes drugs and alters their efficacy and toxicity. Diet alters drugs, the metabolism of the microbiota, and the host. However, whether diet-triggered metabolic changes in the microbiota can alter drug responses in the host has been largely unexplored. Here we show that dietary thymidine and serine enhance 5-fluoro 2′deoxyuridine (FUdR) toxicity in *C. elegans* through different microbial mechanisms. Thymidine promotes microbial conversion of the prodrug FUdR into toxic 5-fluorouridine-5′-monophosphate (FUMP), leading to enhanced host death associated with mitochondrial RNA and DNA depletion, and lethal activation of autophagy. By contrast, serine does not alter FUdR metabolism. Instead, serine alters *E. coli*’s 1C-metabolism, reduces the provision of nucleotides to the host, and exacerbates DNA toxicity and host death without mitochondrial RNA or DNA depletion; moreover, autophagy promotes survival in this condition. This work implies that diet-microbe interactions can alter the host response to drugs without altering the drug or the host.

## Abstract (web summary)

The gut microbiota can alter the effects of anticancer fluoropyrimidines such as 5-fluorodeoxyuridine (FUdR) in the model organism *C. elegans*. Here, the authors show that these effects are further affected by diet, and dietary thymidine and serine increase FUdR toxicity in *C. elegans* via different mechanisms.

## Introduction

Classically, diet has been thought to modulate drug efficacy and toxicity through altering the physiology of the host or by directly interfering with the pharmacodynamics of the drug^1,2^. However, emerging evidence shows that diet can also modulate drug efficacy and toxicity through modifying the composition or physiology of the microbiota, or the interaction between the microbiota and the host^3^. In this study, we utilize a tractable model system to uncover and mechanistically dissect a four-way interaction between the amino acid serine (diet), the chemotherapeutic 5′-fluorodeoxyuridine (drug), the bacterium *E. coli* (microbiota), and the roundworm *C. elegans* (host).

Fluoropyrimidines are commonly used chemotherapeutics, especially for cancers of the GI tract^4^. The most accepted mechanism of action of fluoropyrimidines is inhibition of thymidylate synthase (TS). TS catalyzes the methylation of 2′-deoxyuridine-5′-monophosphate (dUMP) in position 5 of the uracil ring to produce 2′-deoxythymidine-5′-monophosphate (dTMP). TS uses the 1-carbon (1C) metabolite 5,10-methylenetetrahydrofolate (5,10-mTHF) as the indispensable methyl-group donor. TS is critical for cell survival and replication as it is the sole biosynthetic source of dTMP, which is essential for DNA synthesis. When cells are treated in vitro with the fluoropyrimidine 5′-fluorodeoxyuridine (FUdR), they convert FUdR into 5′-fluorodeoxyuridine monophosphate (FdUMP). FdUMP is structurally similar to dUMP, except that it has a fluorine atom in position 5 of the uracil ring. As a consequence, FdUMP forms a stable complex with 5,10-mTHF and TS, preventing the de novo synthesis of dTMP. 5,10-mTHF is essential for dTMP synthesis and for the FdUMP-mediated inhibition of TS^5^. 1C-loaded folates are not known to transfer across membranes; thus, 5,10-mTHF must be locally generated^5^. 5,10-mTHF can be made from the amino acids serine and glycine. Glycine can be degraded via the glycine cleavage system (GCS) to generate NH_3_, CO_2_, and a methyl group that is incorporated into 5,10-mTHF. Separately, the reaction that converts serine to glycine also donates a 1C group to THF to form 5,10-mTHF, which is then available to participate in the methyl transfer reaction that converts dUMP into dTMP. Indeed, 1C units derived from radiolabeled serine are incorporated into nucleotides^6^. Importantly, the levels of 5,10-mTHF are known to limit the efficacy of fluoropyrimidines^4,7^.

Several 1C-metabolites are obtained directly or indirectly from the diet, and the therapeutic value of their dietary supplementation is widely exploited^5^. Serving as a substrate for the synthesis of 5,10-mTHF, the 1C-metabolite folinic acid is the most efficient fluoropyrimidine potentiator^4^. As such, the combination of fluoropyrimidines with folinic acid is a standard treatment for colon cancer^4^. A direct intake route has been delineated for several dietary 1C-metabolites including folates, and serine^5^. By contrast, the potential for bacterial uptake routes for 1C-metabolites has not been given much attention despite evidence in its favor^8^. Studies in mammals show bacterially converted dietary para-aminobenzoate-glutamate—one of the two moieties composing THF—in host tissues^9,10^, *C. elegans* studies demonstrate that *E. coli* mediates the effect of dietary supplementation of folic acid on lifespan^11^, and mouse studies show that bacterially derived serine can affect kidney function^12^. Given that dietary 1C-metabolites, such as folinic acid, are among the most effective potentiators of fluoropyrimidine action, and that the microbiota can alter dietary 1C-metabolites or produce them from dietary precursors, four-way interactions between dietary folates or their precursors, fluoropyrimidines, microbes, and the host, could modulate fluoropyrimidine efficacy and/or toxicity in vivo. In the past several years, *C. elegans* has been exploited as a model system to study complex drug–microbe–host interactions. Garcia et al.^13^ and Scott et al.^14^ developed a three-way drug–microbe–*C. elegans* system revealing that microbes mediate chemotherapeutic efficacy in *C. elegans*. More recently, Pryor et al.^15^ developed a host–microbe–drug-dietary nutrient screen to study the interaction between *C. elegans*, *E. coli*, the biguanide metformin, and dietary nutrients. Here we independently developed three and four-way screening strategies to identify and mechanistically dissect the four-way interactions that modulate FUdR toxicity in *C. elegans*.

First, we investigate the mechanism of toxicity underlying the three-way interaction between FUdR, *E. coli*, and *C. elegans*. On the microbe side, we validate that conversion of FUdR into 5-fluorouridine monophosphate (FUMP), and not dTMP depletion, contributes to toxicity in *C. elegans*. On the host side, we define that FUdR toxicity (likely via worm-derivatives of FUMP) targets mitochondrial RNAs and DNA, and that *C. elegans* die from activation of a lethal mitochondria-to-autophagy axis. Then, we investigate the four-way interaction between dietary metabolites, FUdR, *E. coli*, and *C. elegans*. We show that dietary supplementation with thymidine or serine transforms sublethal doses of FUdR (no apparent toxicity) into lethal ones (100% embryonic lethality) through altering the metabolism of the microbe. However, the mechanisms of action of thymidine and serine are distinct. Thymidine simply enhances the mechanisms driving the three-way interaction, whereas serine acts via enabling dTMP depletion in *E. coli* and consequently in the host. Most strikingly, dietary serine redefines, or even reverts, the role that host pathways have on executing FUdR toxicity, unveiling sub-phenotypic complexity in four-way diet–drug–microbiota–host interactions.

## Results

### FUdR toxicity due to *E. coli* FUMP synthesis, not dTMP depletion

To define whether and how dietary nutrients alter the toxicity of FUdR in *C. elegans*, it is required first to identify the minimum dose leading to robust toxicity (i.e. 100% embryonic lethality) for further screens on dietary enhancers and inhibitors of the toxicity. We identified 1 ± 0.25 µg/mL FUdR as the dose causing 100% embryonic lethality when worms were cultured on *E. coli* BW25113 (parental strain of all *E. coli* mutants used in this study), and 7.5 ± 2.5 µg/mL FUdR as the dose causing 100% embryonic lethality when worms were cultured on *E. coli* HB101 (parental strain of all *C. elegans* RNAi clones used in this study). We hereinafter refer to these doses as Lth-FUdR (for Lethal FUdR) (Supplementary Fig. 1a).

**Fig. 1:**
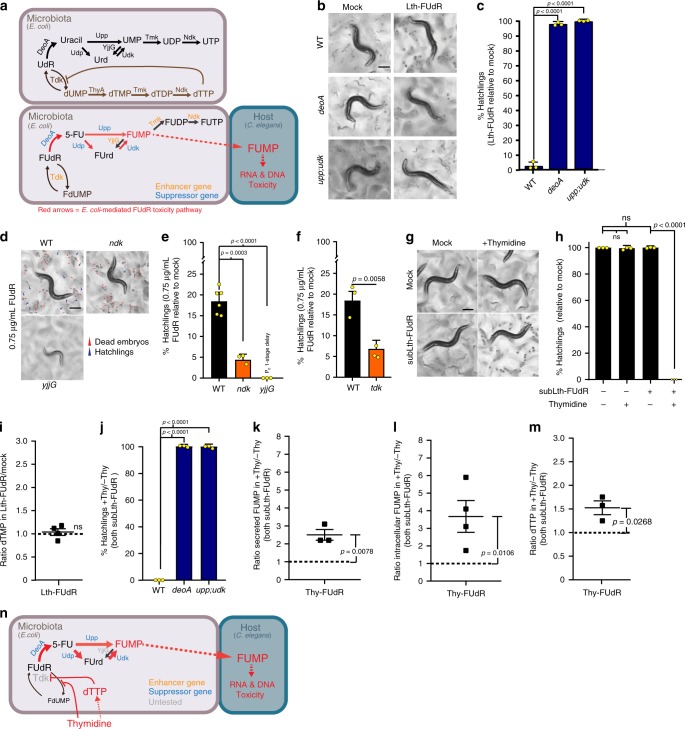
Dietary thymidine enhances FUdR-to-FUMP conversion in *E. coli*. Throughout this figure: % hatchlings is estimated as [live hatchlings/(live hatchlings + live embryos + dead embryos)] in the condition of interest relative to % hatchlings in mock of the same *E. coli* or *C. elegans* genotype; ≥5 images per treatment were quantitated; statistical significance was assessed via two-tailed unpaired nonparametric *t*-test. LC–MS data was analyzed using one-tailed ratio *t*-test after ROUT outlier treatment. Data are presented as mean values ± SEM, scale bars = 200 µm, *n* = # independent biological replicates. Source data are provided as a Source Data file. **a** Top: endogenous pyrimidine ribonucleotide salvage pathway (black font) and dTMP de novo synthesis pathway (brown font). Bottom: Model of *E. coli*-mediated FUdR-to-FUMP toxicity pathway. **b** Representative images of progeny viability of *C. elegans* treated with mock or Lth-FUdR while cultured on WT (BW25113), *deoA*, or *upp;udk* KO *E. coli* lawns. **c** Quantification of **b** treatments. *n* = 3. **d** Representative images of progeny viability of *C. elegans* treated with mock or 0.75 μg/mL FUdR (lower dose to detect enhancers) while cultured on WT (BW25113), *ndk*, or *yjjG* KO *E. coli* lawns. **e** Quantification of panel **d** treatments. *n* = 3. **f** Quantification of progeny viability of *C. elegans* treated with mock or 0.75 μg/mL FUdR while cultured on WT (BW25113) or *tdk* KO *E. coli* lawns. *n* = 3. **g** Representative images of progeny viability of *C. elegans* treated with FUdR (0.25 μg/mL) ± 5 mg/mL thymidine. **h** Quantification of **g** treatments. *n* = 3. **i** LC–MS measurement of dTMP normalized to [^13^C9,^15^N2]UMP in *E. coli* treated with Lth-FUdR (5 µg/mL) relative to mock. *n* = 4. **j** Quantification of progeny viability of *C. elegans* cultured on WT (BW25113), *upp;udk*, or *deoA* KO *E. coli* lawns treated with subLth-FUdR (0.25 μg/mL) ± 5 mg/mL thymidine. *n* = 3. **k** LC–MS measurement of secreted FUMP in *E. coli* supernatants normalized to [^13^C9,^15^N2]UMP. *n* = 3. **l** LC–MS measurement of FUMP normalized to [^13^C9,^15^N2]UMP in *E. coli* pellets, *n* = 4. **m** LC–MS measurement of dTTP normalized to [13C9,15N2]UMP in *E. coli* pellets, *n* = 3. **n** Working model of *E. coli*-mediated thymidine-enhanced FUdR toxicity: (1) thymidine-derived dTTP inhibits Tdk, and (2) dietary thymidine competes with FUdR, thereby promoting FUdR-to-FUMP bioconversion.

We then moved on to defining the mechanism of toxicity of Lth-FUdR using a three-way FUdR–*E. coli*–*C. elegans* high-throughput screening strategy (summarized in Fig. 1a). We found that KO of *E. coli deoA* suppresses Lth-FUdR toxicity in *C. elegans* (Fig. 1b, c). DeoA can convert FUdR into Fluorouracil (5-FU) (Fig. 1a). Scott et al.^14^ demonstrated that 5-FU is also a prodrug that needs to be converted to be toxic to *C. elegans*. Hence, *E. coli* DeoA likely carries out one of multiple steps in the conversion of FUdR into the actual toxic derivatives. A reasonable hypothesis would be that the toxic derivative that *E. coli* produces is FUMP, as this would be in line with genetic evidence presented by Garcia et al.^13^. However, single KO of *E. coli upp*, *udp*, or *udk* was not sufficient to suppress Lth-FUdR toxicity in our screen or follow up retesting (Supplementary Fig. 1b, c). As *upp* and *udk* encode for redundant enzymes capable of converting 5-FU into FUMP, we tested a double KO. Indeed, double KO of *E. coli upp* and *udk* completely suppresses Lth-FUdR toxicity in *C. elegans* (Fig. 1b, c). These results suggest that *E. coli* uses the pyrimidine ribonucleotide salvage pathway (i.e. FUdR-to-FUMP conversion pathway) to convert the prodrug FUdR into a derivative toxic to *C. elegans*. Because nucleotide polyphosphates may not be efficiently taken up by the host, FUMP would be more likely than its downstream derivatives FUDP or FUTP to be the toxic derivative that *E. coli* produces and *C. elegans* takes up. To approximate an answer to this question, we supplemented the plates with UMP (the non-fluorinated analog of FUMP), or the UMP precursors uridine and uracil. We found all three compounds to rescue Lth-FUdR toxicity in *C. elegans*. By contrast, supplementation with UDP or UTP does not rescue the toxicity (Supplementary Fig. 1d). These results are in line with the notion that nucleotide monophosphates or their unphosphorylated precursors can cross membranes, and hence, could be taken up by the *C. elegans* host while nucleotide polyphosphates would not, and suggest that *E. coli*-generated nucleotide polyphosphates may not be significant contributors to *E. coli*-mediated FUdR toxicity in *C. elegans*. In further support of this notion, chemical inhibition of Tmk (the *E. coli* enzyme that would produce FUDP) further enhances (instead of suppressing) Lth-FUdR toxicity (Supplementary Fig. 1e). Similarly, KO of *ndk* (the *E. coli* gene encoding the enzyme that would produce FUTP) enhances FUdR toxicity (Fig. 1d, e). Together, the data argue against *E. coli*-generated FUDP or FUTP being significant contributors to FUdR toxicity in *C. elegans*. Another *E. coli*-generated and potentially toxic derivative of FUdR is 5′-fluorouridine (FUrd). However, KO of *yjjG* (the *E. coli* gene encoding the enzyme that produces FUrd) enhances FUdR toxicity in *C. elegans* (Fig. 1d, e). Furthermore, the *upp;udk* double KO (2KO) and the *upp;udk;udp* triple KO (3KO) both rescue Lth-FUdR toxicity to the same extent (Supplementary Fig. 1c, f). This result argues against FUrd being a significant contributor to *E. coli*-mediated FUdR toxicity, because in the 2KO, FUrd synthesis is favored due to 5-FU to FUrd conversion, whereas in the 3KO, such conversion is blocked (pathway scheme in Fig. 1a).

Finally, a major candidate to be an *E. coli*-generated mediator of the toxicity is FdUMP. *E. coli*-generated FdUMP could act via: (1) inhibiting *C. elegans* TS post-ingestion; or (2) inhibiting *E. coli* TS and consequently reducing the availability of thymidine in the *C. elegans* diet because *E. coli* is the main source of nucleotides for *C. elegans*^16^. An essential step for both mechanisms of action is that *E. coli* thymidylate kinase (Tdk) converts FUdR into FdUMP. The result of the screen and the follow up retesting showing that KO of *tdk* enhances, instead of suppressing, Lth-FUdR toxicity (Fig. 1f) argues against an FdUMP-dependent mechanism of action. In the same line, thymidine supplementation enhances, instead of rescues, Lth-FUdR toxicity (Fig. 1g, h), and LC–MS analyses show that the levels of dTMP did not drop in *E. coli* treated with a lethal dose of FUdR (Fig. 1i). Thus, the evidence argues against FdUMP directly produced by *E. coli*, or dTMP depletion in *E. coli* contributing to Lth-FUdR toxicity in *C. elegans*.

In summary, when FUMP synthesis is blocked, we observe abrogation of the toxicity, and when FUMP synthesis or accumulation is promoted, we observe enhanced toxicity. In addition, blocking FdUMP synthesis (*tdk* KO) enhances the toxicity. Therefore, our data support a model in which FUMP would be the major link between microbe and host fluoropyrimidine metabolism, and host-generated derivatives of FUMP (e.g. FUTP or FdUTP) would promote toxicity in the host.

### Thymidine increases *E. coli*-mediated FUdR to FUMP conversion

The observation that in-plate supplementation with thymidine increases FUdR toxicity in *C. elegans* and that FUdR toxicity in *C. elegans* is mediated by *E. coli*, suggest that dietary thymidine, FUdR, *E. coli*, and *C. elegans* may represent an uncharacterized four-way diet–drug–microbe–host interaction. To test the hypothesis that *E. coli* is mediating the potentiating effect of thymidine, we tested whether *E. coli* pretreated with a sublethal dose of FUdR (subLth-FUdR) plus thymidine would be more toxic to *C. elegans* than *E. coli* pretreated with subLth-FUdR alone. In this context, worms were not directly exposed to FUdR or thymidine; hence, enhanced toxicity would support the hypothesis that thymidine-enhanced FUdR toxicity is bacterially driven (Experimental setup in Supplementary Fig. 2a). In addition, because the known mechanism of 5-FU toxicity is production and secretion of FUMP^14^, we separated and independently tested the supernatants and pellets of *E. coli* pretreated with subLth-FUdR plus thymidine. Finally, the filter-sterilized supernatants were seeded on top of triple *upp,udp,udk* KO lawns to avoid in-plate bacterially driven conversion of the FUdR remaining in the *E. coli* supernatants. We observed that the supernatants and the pellets of *E. coli* pretreated with Lth-FUdR and subLth-FUdR plus thymidine caused embryonic lethality, while the supernatants and pellets pretreated with subLth-FUdR or thymidine alone were not toxic to *C. elegans* (Supplementary Fig. 2b). Therefore, thymidine-enhanced FUdR toxicity (TE-FUdR) is bacterially driven and mediated, at least in part, by a secretable toxic compound. We first tested whether this secretable toxic compound would be the *E. coli*-generated FUdR-derivative FUMP. In support of this hypothesis, KO of the gene encoding the *E. coli* enzymes capable of converting FUdR into FUMP (*deoA*, or double KO of *upp* and *udk*) suppresses TE-FUdR toxicity (Fig. 1j). More directly, we found a >2 fold increase in FUMP levels when we compare the supernatants and bacterial pellets of *E. coli* treated with FUdR plus thymidine relative to FUdR alone (Fig. 1k, l). Therefore, dietary thymidine enhances FUdR toxicity in the *C. elegans* host through promoting FUdR-to-FUMP conversion via the pyrimidine ribonucleotide salvage pathway.

**Fig. 2:**
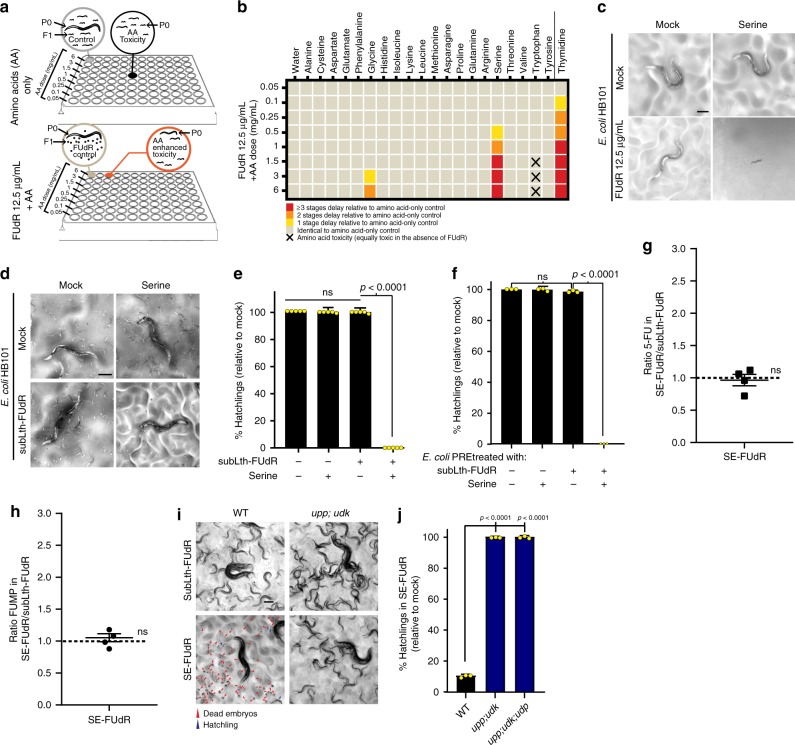
Dietary serine enhances FUdR toxicity but not FUdR-to-FUMP conversion. Throughout this figure: % hatchlings and LC–MS data were analyzed as described in Fig. 1. Statistical significance was assessed via two-tailed unpaired nonparametric *t*-test for % hatchlings quantification. LC–MS data were analyzed using one-tailed ratio *t*-test after ROUT outlier treatment. Data are presented as mean values ± SEM, scale bars = 200 µm, *n* = # independent biological replicates. Source data are provided as a Source Data file. **a** Screen setup to search for dietary amino acids (AAs) that enhance FUdR toxicity (cause developmental delay). Worms cultured on *E. coli* HB101 were treated with 12.5 µg/mL FUdR from the L1 stage ± serial dilutions of AAs (0.05–6 mg/mL). AA-only wells were included to detect AA toxicity. Wells were scored after 60 h at 20 °C, when FUdR-only wells show 100% embryonic lethality but no developmental delay. **b** Heat map representing degree of developmental delay caused by supplemented AAs. Color and symbol key depicted below. Thymidine is a positive control. **c** Representative images of targeted validation of developmental delay induced by co-administration of 12.5 µg/mL FUdR with 1.5 mg/mL of serine. Images taken after 60 h of incubation at 20 °C. *n* > 3. **d** Representative images of progeny viability of *C. elegans* cultured on HB101 and treated from L1 with mock, subLth-FUdR (1 μg/mL FUdR, which is sublethal because using HB101), 1.5 mg/mL serine, or subLth-FUdR plus serine. *n* > 10. **e** Quantification of % hatchlings relative to mock of treatments represented in **d**. *n* = 5. **f** Quantification of % hatchlings in worms exposed to lawns of *E. coli* pretreated “in tube” with ±subLth-FUdR ±serine. In this setup, worms are not directly exposed to FUdR or serine (Supplementary Fig. 4). *n* = 3. **g** LC–MS measurement of intracellular 5-FU relative to internal standard (IS) [1,3-^15^N2]Uracil in *E. coli* treated with subLth-FUdR plus serine compared to subLth-FUdR. *n* = 4. **h** LC–MS measurement of intracellular FUMP relative to internal standard (IS) [^13^C9,^15^N2]UMP in *E. coli* treated with subLth-FUdR plus serine compared to subLth-FUdR. n = 4. **i** Representative images of progeny viability of *C. elegans* cultured on WT (BW25113) or *upp;udk* KO *E. coli* lawns treated from L1 with subLth-FUdR (0.25 μg/mL) ± serine. **j** Quantification of % hatchlings relative to mock of treatments represented in **i** and the triple *E. coli* KO *upp;udp;udk*. *n* = 3.

We then asked how thymidine potentiates the toxicity of FUdR. Clues came from the following: (1) KO of the gene encoding Tdk, the enzyme that can convert FUdR into FdUMP, enhances FUdR toxicity (Fig. 1f). This likely occurs because by blocking the conversion of FUdR into FdUMP, we favor the conversion of FUdR into 5-FU and then FUMP (pathway scheme in Fig. 1a); (2) Tdk accepts thymidine as a substrate (ecocyc.org). Hence, thymidine can compete with FUdR and reduce the Tdk-mediated conversion of FUdR into FdUMP; and (3) Tdk is subject to end-product inhibition by dTTP (ecocyc.org). As thymidine can serve as a substrate for the synthesis of dTTP, then dietary thymidine could promote end-product inhibition of Tdk. In support of the latter mechanism (but without ruling out the former), we observed increased levels of dTTP in TE-FUdR *E. coli* (Fig. 1m). Together, the data are consistent with dietary thymidine increasing the toxicity of FUdR via indirectly promoting the conversion of FUdR into FUMP (Working model in Fig. 1n).

### Serine increases FUdR toxicity without increasing FUMP levels

After establishing that four-way diet–drug–*E. coli*–*C. elegans* interactions such as the thymidine–FUdR–*E. coli*–*C. elegans* interaction can be detected and mechanistically dissected in our system, we sought to identify common dietary nutrients that may affect FUdR toxicity. We focused on amino acids (AA) for four reasons: (1) AA derivatives are precursors for the synthesis of nucleotides and cofactors needed to synthesize nucleotides^17^; (2) AAs alter chemotherapeutic efficacy in cells in vitro^18^; (3) AAs are among the most highly consumed nutrients by cancer cells^19^; and (4) AA-depleted diets are currently being tested to improve cancer treatment^20^. We tested 19 l-amino acids and glycine for their capacity to promote developmental delay of *C. elegans* treated with a dose of FUdR that on its own does not affect development. The conditions of the four-way compound screen are depicted in Fig. 2a and are described in the Methods section. Of the 20 AA, we found that high doses of tryptophan were toxic on their own whereas glycine and serine increased toxicity in a FUdR-specific manner (Fig. 2b). Serine was a stronger toxicity potentiator than glycine, so we focused on characterizing serine-enhanced FUdR toxicity. First, we retested the capacity of serine to potentiate the toxicity of an already lethal dose of FUdR. Hatchlings seeded on *E. coli* HB101 lawns supplemented with 1.5 mg/mL serine alone became fertile adults after 60 h of incubation at 20°C, while hatchlings parallelly growing on 12.5 µg/mL FUdR were sterile adults. However, when we combined serine and FUdR, we observed larval arrest that persisted indefinitely (Fig. 2c), demonstrating that dietary supplementation of serine can potentiate the toxicity of FUdR. Furthermore, hatchlings seeded on *E. coli* HB101 lawns supplemented with 1.5 mg/mL serine or a sublethal dose of FUdR (1 µg/mL FUdR for HB101) yielded 100% fertile adult *C. elegans*; however, worms were 100% sterile when serine and FUdR were combined (Fig. 2d, e). Together, the results show that dietary supplementation of serine can potentiate the toxicity of FUdR across a wide range of doses and toxicity outcomes. Hereafter, we use the term SE-FUdR toxicity to refer to the enhancement of toxicity achieved by combining a sublethal dose of FUdR (≤0.25 µg/mL for BW25113 or ≤1 µg/mL for HB101) with dietary serine (Supplementary Fig. 3a).

How does dietary serine enhance FUdR toxicity in *C. elegans*? A formal possibility is that combining FUdR with serine impairs *E. coli* growth, thus leading to food scarcity in the worm. However, for all non-screening experiments presented in this study, bacteria were cultured overnight in LB in the absence of additives, and then washed and concentrated in S-buffer to OD_600nm_ = 20 before being seeded on nematode growth media (NGM) plates. Furthermore, CFU counting of bacteria harvested from standard NGM or NGM supplemented with serine, subLth-FUdR, or subLth-FUdR plus serine shows similar bacterial viability in all conditions (Supplementary Fig. 3b, c). Therefore, *E. coli* lawn density and growth rates do not appear to explain SE-FUdR toxicity in our experimental setup.

We then tested whether SE-FUdR toxicity, like thymidine, was bacterially driven. As with thymidine, we pretreated liquid cultures of *E. coli* with mock, Lth-FUdR, serine, subLth-FUdR, or subLth-FUdR plus serine, and separated and tested *E. coli* supernatants and pellets independently (Experimental setup in Supplementary Fig. 4a). We observed toxicity in worms cultured on *E. coli* pellets pretreated with SE-FUdR (Fig. 2f and Supplementary Fig. 4b). However, we observed no toxicity in worms exposed to supernatants of *E. coli* pretreated with SE-FUdR (Supplementary Fig. 4b), demonstrating that, unlike thymidine, SE-FUdR toxicity is not driven by secreted *E. coli* products, and justifying to not further characterize SE-FUdR *E. coli* supernatants in this study. Therefore, SE-FUdR toxicity is bacterially driven, but mainly via an intracellular mechanism. Based on this observation, we can formulate two hypotheses: (1) serine promotes FUdR-to-FUMP conversion but prevents FUMP secretion; or (2) serine promotes a mechanism of toxicity that is distinct from Lth-FUdR and TE-FUdR toxicity. To test the first hypothesis, we measured the levels of 5-FU and FUMP in the *E. coli* pellets, and found them to be the same in the subLth-FUdR and SE-FUdR conditions (Fig. 2g, h), even though aliquots of the bacteria used for metabolite extraction showed the expected 0 and 100% embryonic lethality, respectively (Supplementary Fig. 5a). This lack of increase of FUMP levels in the SE-FUdR condition is in contrast with the elevated levels of FUMP observed in the TE-FUdR condition (Fig. 1k, l), and supports the notion that thymidine and serine potentiate FUdR toxicity through distinct mechanisms.

As the SE-FUdR mechanism of toxicity seems distinct from Lth-FUdR and TE-FUdR, we tested whether fluororibonucleotides other than FUMP, specifically fluorouridine (FUrd), FUDP, or FUTP, were contributing to SE-FUdR. These fluororibonucleotides were below the detection limit of our LC–MS of bacteria or worms treated with subLth-FUdR or SE-FUdR (Supplementary Fig. 5b, and experimental details in Supplementary Note 1). Nevertheless, KO of *yjjG* or *udp*, which would reduce FUrd synthesis (pathway scheme in Fig. 1a), does not reduce SE-FUdR toxicity (Supplementary Fig. 5c). Furthermore, blocking the conversion of FUMP-into-FUDP (through chemical treatment with 5′-iodo-UMP) and of FUDP-into-FUTP (through KO of *ndk*) further enhances SE-FUdR toxicity, arguing against FUDP or FUTP mediating SE-FUdR toxicity (Supplementary Fig. 5d, e, respectively). Remarkably, these data show that despite the fact that SE-FUdR toxicity is not driven by increased FUMP (Fig. 2h), preventing the conversion of FUMP into FUDP or FUTP further enhances SE-FUdR toxicity. These results prompted us to think about how SE-FUdR toxicity and the FUdR-to-FUMP toxicity pathway interact. We hypothesized that sublethal levels of FUMP toxicity would be necessary to sensitize *C. elegans* to SE-FUdR toxicity. In support of this hypothesis, we found that the double KO *upp;udk*, and the triple KO *upp;udp;udk* suppress SE-FUdR toxicity in *C. elegans* (Fig. 2i, j). Altogether, the results suggest that SE-FUdR is not mediated by increased conversion of FUdR into FUMP (biochemical evidence), or FUDP, FUTP or FUrd (genetic evidence) in *E. coli*. However, a sublethal level of FUMP toxicity appears to be required to sensitize *C. elegans* to SE-FUdR toxicity.

### *E. coli*’s folate metabolism is required for SE-FUdR toxicity

**Fig. 3:**
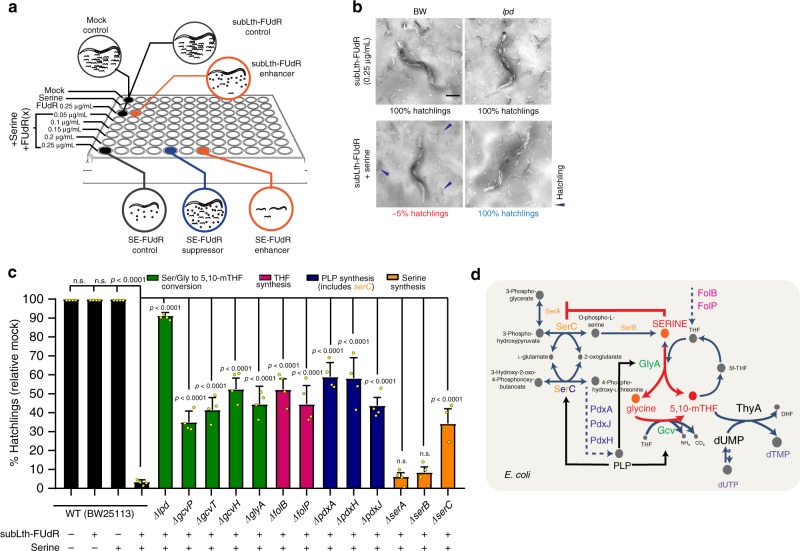
SE-FUdR toxicity requires *E. coli*’s folate and pyridoxal phosphate synthesis pathways. Throughout this figure: statistical significance was assessed via two-tailed unpaired nonparametric *t*-test for % hatchlings quantification. Data are presented as mean values ± SEM, scale bars = 200 µm, *n* = # independent biological replicates. Source data are provided as a Source Data file. **a** Setup of four-way *E. coli* KO screen for mediators of serine-enhanced FUdR toxicity. Screening was carried out in the BW25113 background in triplicate at 25 °C in 8 conditions: (1) mock, (2) serine 1.5 mg/mL, (3) subLth-FUdR 0.25 µg/mL, and (4–8) serine 1.5 mg/mL plus subLth-FUdR from 0.05 to 0.25 µg/mL (lower doses included to detect toxicity enhancers). Each column of the 96-well plate corresponds to a different *E. coli* KO. Developmental stage and progeny viability were scored. **b** Representative images of validation of the toxicity-suppressor effect of knocking down *E. coli lpd*, making evident that SE-FUdR toxicity is bacterially driven. *n* = 4. **c** Effect of the *E. coli* suppressors of SE-FUdR toxicity on progeny viability (% hatchlings). Images and data were analyzed as described in Fig. 1. *n* = 4. **d** Working model of how dietary serine promotes synthesis of 5,10-mTHF in *E. coli*. Color codes of suppressor gene names are consistent with **c**. Serine relevant actions (depicted in red): (1) inhibits its own synthesis releasing *serC* to promote PLP synthesis (PLP is an essential cofactor for GlyA and the GCV complex); and (2) serves as a substrate for the synthesis of 5,10-mTHF directly via GlyA and indirectly via the GCV complex.

Having ruled out enhanced FUdR-to-FUMP conversion, we moved to uncover the main bacterially driven mechanism of SE-FUdR toxicity. First, we tested whether in-plate supplementation with serine would simply increase the levels of serine in *E. coli*, and hence, although bacterially driven, SE-FUdR would not require *E. coli*-mediated conversion of serine. Arguing against this notion, direct measurement of serine and glycine levels in *E. coli* and *C. elegans* shows no increase in the levels of these amino acids (Supplementary Fig. 5f, g), even when aliquots of the analyzed bacteria promote enhanced toxicity in the worm (Supplementary Fig. 5a). Hence, we decided to use a four-way *E. coli* KO suppressor/enhancer screen to molecularly dissect how serine is metabolized in *E. coli* to enhance FUdR toxicity in *C. elegans*. To create our *E. coli*-KO screening library, we used in silico modeling based on the iJO1366 *E. coli* metabolic model to search for all *E. coli* genes within two-metabolic steps from the homologs of the mammalian fluoropyrimidine metabolic pathways (gene list in Supplementary Table 1, and 96-well screen setup in Fig. 3a). The four-way high-throughput screen identified 29 *E. coli* genes altering SE-FUdR toxicity in *C. elegans* (Supplementary Table 2). Genes belonging to metabolic pathways enriched among the hits were retested in 6 cm NGM plates. Twelve primary hits were validated using the following criteria: (1) > or <10% hatchlings than worms seeded on WT *E. coli*; (2) *p*-value < 0.05; and (3) growth of the *E. coli* KO clone in ±serine ±FUdR did not correlate with its enhancer or suppressor phenotype (Supplementary Fig. 6). Among the SE-FUdR toxicity suppressors, *lpd* emerged as the strongest hit (Fig. 3b, c). Lpd encodes *E. coli* lipoamide dehydrogenase, which is part of three multicomponent enzymatic complexes: pyruvate dehydrogenase, 2-oxoglutarate dehydrogenase, and the glycine cleavage complex (GCV). The GCV complex is composed of Lpd, GcvP, GcvT, and GcvH, and catalyzes the conversion of glycine into 5,10-methylene-tetrahydrofolate (5,10-mTHF). KO of *gcvP*, *gcvT*, and *gcvH*, also suppresses SE-FUdR toxicity (Fig. 3c), favoring the notion that Lpd would contribute to SE-FUdR toxicity as a component of the GCV complex. However, *lpd* is a stronger suppressor than the *gcv* genes. Several distinctions exist between Lpd and the Gcv proteins. For instance, the *gcv* genes are part of a single operon transcribed by Fnr, whereas *lpd* is encoded as a single gene and is transcribed by Crp (ecocyc.org). In addition, Lpd is necessary for the activation of the Gcv proteins^21^. However, given the stronger suppressor phenotype of *lpd* relative to the *gcv* genes, roles for Lpd beyond the GCV complex cannot be ruled out. Nevertheless, KO of *glyA*, which encodes the enzyme that converts serine into glycine and 5,10-mTHF, also suppresses SE-FUdR toxicity (Fig. 3c, and pathway scheme in Fig. 3d). Together, GlyA and the GCV complex can convert serine into 5,10-mTHF (pathway scheme in Fig. 3d); and therefore, these suppressors suggest that 5,10-mTHF may have an important role in mediating SE-FUdR toxicity. Pointing to the same direction, KO of *folP* and *folB*, suppresses SE-FUdR toxicity (Fig. 3c). FolP and FolB synthesize tetrahydrofolate, which is the precursor of 5,10-mTHF (pathway scheme in Fig. 3d). Further, both the GCV complex and GlyA require the cofactor vitamin B6 (pyridoxal-5′-phosphate or PLP) to synthesize 5,10-mTHF (ecocyc.org). Hence, it is relevant that KO of the PLP-synthesis genes *pdxA, pdxJ, pdxH*, and *serC* suppress SE-FUdR toxicity (Fig. 3c). Of note, the other enzymatic complexes containing Lpd, namely pyruvate dehydrogenase and 2-oxoglutarate dehydrogenase, do not use PLP as a cofactor, further favoring the notion that the main contribution of Lpd to SE-FUdR toxicity would be through its role as a component of the GCV complex.

Additional insight into how serine potentiates the toxicity of FUdR is garnered from the role that the serine-synthesis pathway has in SE-FUdR toxicity. SerA, SerB, and SerC are essential for de novo synthesis of serine in *E. coli* (ecocyc.org). However, only KO of *serC* suppresses SE-FUdR toxicity in *C. elegans* (Fig. 3c). Distinctively, SerC is involved in PLP synthesis, whereas SerA and SerB only contribute to serine synthesis (pathway scheme in Fig. 3d). Also importantly, SerA is subject to end-product inhibition by serine. Therefore, the data suggest that serine promotes SE-FUdR toxicity via promoting the synthesis of 5,10-mTHF. Serine would promote 5,10-mTHF synthesis through at least two mechanisms (Fig. 3d): (1) inhibiting its own synthesis (via SerA inhibition), thereby freeing SerC to synthesize PLP; and (2) serving as a substrate for the synthesis of 5,10-mTHF via GlyA and the GCV complex. The capacity to free SerC via end-product inhibition distinguishes serine from glycine, and could underlie the observation that dietary glycine is a weaker potentiator of FUdR toxicity than serine (Fig. 2b). Altogether, dietary serine promotes FUdR toxicity in *C. elegans* through a bacterially driven mechanism that involves conversion of serine and glycine into 5,10-mTHF, and not increased bacterial conversion of FUdR-into-FUMP or accumulation of serine or glycine.

### Dietary serine reduces *E. coli*’s and hence *C. elegans*’ dTMP pool

**Fig. 4:**
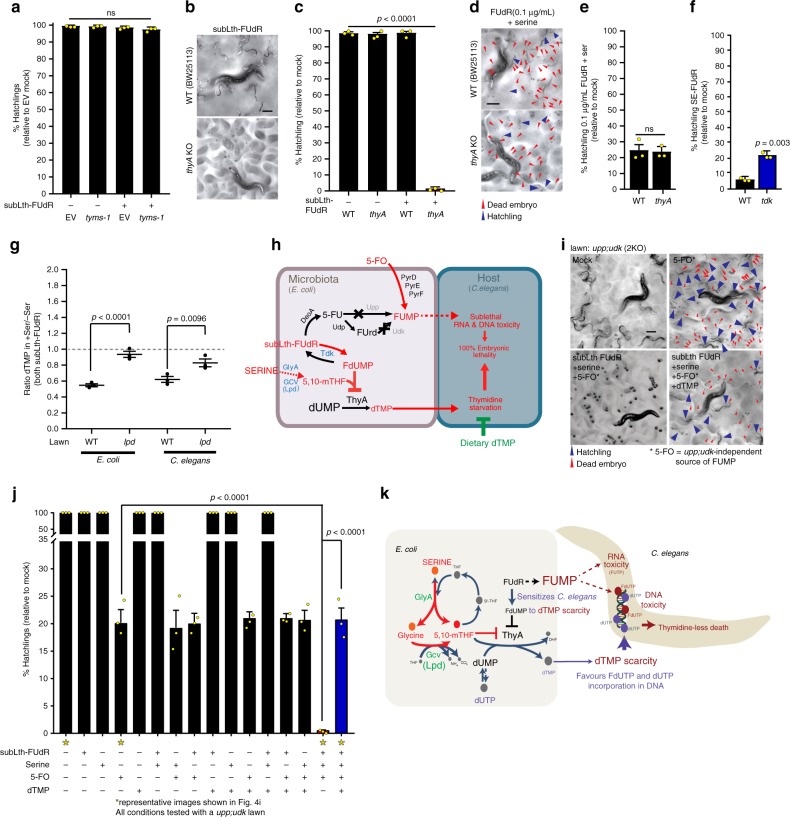
SE-FUdR promotes dTMP depletion in *E. coli* and *C. elegans*. Throughout this figure: % hatchlings and LC–MS data were analyzed as described in Fig. 1. Statistical significance was assessed via two-tailed unpaired nonparametric *t*-test for % hatchlings quantification. LC–MS data were analyzed using one-tailed ratio *t*-test after ROUT outlier treatment. Data are presented as mean values ± SEM, scale bars = 200 µm. *n* = # independent biological replicates. Source data are provided as a Source Data file. **a** Quantification of progeny viability of *C. elegans* exposed to subLth-FUdR (1 μg/mL) while cultured on EORB1 (RNAi-competent derivative of HB101) carrying empty RNAi empty vector (EV) or RNAi against *tyms-1*. *n* = 3. See EORB1 strain development in Methods and Supplementary Fig. 8. **b** Representative images of progeny viability of *C. elegans* exposed to subLth-FUdR (0.25 μg/mL) while cultured on WT (BW25113) or *thyA* KO *E. coli* lawns. **c** Quantification of **b** treatments. *n* = 3. **d** Representative images of progeny viability of *C. elegans* exposed to subLth-FUdR (0.1 μg/mL) + serine 1.5 mg/mL while cultured on WT (BW25113) or *thyA* KO *E. coli* lawns. **e** Quantification of **d** treatments. *n* = 3. **f** Quantification of progeny viability of *C. elegans* exposed to subLth-FUdR ± serine while cultured on WT (BW25113) or *tdk* KO *E. coli* lawns. *n* = 3. **g** LC–MS measurement of dTMP normalized to [^13^C9,^15^N2]UMP (Norm dTMP) in *E. coli* WT (BW25113) and *lpd* KO, and *C. elegans* cultured in these two *E. coli* strains. The ratio Norm dTMP in SE-FUdR / Norm dTMP in subLth-FUdR is depicted for each treatment. *n* = 3. **h** Setup of dTMP-rescue experiment: (1) *upp;udk* double KO lawn avoids enhanced-FUMP toxicity otherwise driven by thymidine; (2) 5′-fluoroorotic acid (5-FO) as a source of FUMP; (3) subLth-FUdR + serine to promote SE-FUdR; (4) ±dTMP to test rescue of SE-FUdR toxicity. **i** Representative images of progeny viability of *C. elegans* cultured on *upp;udk* double KO *E. coli* lawns ±5-FO, ±SE-FUdR (0.25 μg/mL FUdR and 1.5 mg/mL serine), ±dTMP (1.5 μg/mL) showing dTMP rescues SE-FUdR toxicity. **j** Quantification of treatments in **i** (denoted with asterisks) and other controls. *n* = 3. **k** Working model of SE-FUdR toxicity. Through promoting 5,10-mTHF synthesis, dietary serine enables FdUMP-mediated inhibition of *E. coli* TS (ThyA). The consequent scarcity of dietary dTMP then exacerbates the toxic effect of sublethal FUdR, leading to DNA toxicity, and death of the worm.

The observation that 5,10-mTHF synthesis in *E. coli* is essential to SE-FUdR toxicity points toward the best characterized mechanism of fluoropyrimidine toxicity: the formation of a ternary complex composed of FdUMP, 5,10-mTHF, and thymidylate synthase (TS) that inhibits TS function^4^. Importantly, mammalian evidence suggests that 5,10-mTHF is the main limiting factor in the formation of this inhibitory complex^4^. Hence, the next step was to define whether *E. coli*-generated 5,10-mTHF might act through inhibition of the worm TS or *E. coli* TS, or both. If worm TS is inhibited, serine would promote 5,10-mTHF synthesis in *E. coli*, elevating the levels of 5,10-mTHF in the *C. elegans* diet, and enabling the inhibition of *C. elegans*’ TS (TYMS-1). Arguing against this scenario, 5,10-mTHF is known to poorly cross membranes^5^, and strong reduction of *C. elegans* TS expression through RNAi against *C. elegans tyms-1* (Supplementary Fig. 7a) does not enhance FUdR toxicity (Fig. 4a). Using a similar rationale, if dietary serine enhances FUdR toxicity mainly by enabling the inhibition of *E. coli*’s TS, culturing worms on TS-deficient *E. coli* (*thyA* KO) should enhance their sensitivity to sublethal doses of FUdR. Indeed, although feeding *thyA* KO bacteria or treating with subLth-FUdR alone is not toxic to *C. elegans*, feeding *thyA* KO bacteria in the presence of sublethal levels of FUdR leads to >90% embryonic lethality in *C. elegans* (Fig. 4b, c), phenocopying SE-FUdR. Furthermore, the enhanced FUdR toxicity observed in worms fed the *thyA* KO cannot be further enhanced by dietary serine (Fig. 4d, e), suggesting that *thyA* KO and dietary serine enhance FUdR toxicity through the same mechanism (see rationale of conditions for this experiment in Supplementary Note 2). Another prediction of the scenario in which dietary serine enables the inhibition of *E. coli*’s TS is that SE-FUdR toxicity would depend upon *E. coli* capacity to convert FUdR into FdUMP, a reaction carried out by Tdk. In line with this prediction, we found that KO of *E. coli tdk* partially suppresses SE-FUdR toxicity (Fig. 4f). The observed modest suppression is expected because KO of *tdk* would simultaneously enhance FUdR-to-FUMP bioconversion (Fig. 1f and pathway scheme in Fig. 1n).

The above observations are in line with a model in which dietary serine, via promoting the synthesis of 5,10-mTHF, enables the inhibition of *E. coli* ThyA, thereby reducing the levels of dTMP in the *C. elegans* diet. To test this model directly, we measured dTMP levels in *E. coli*. We found that *E. coli* treated with subLth-FUdR plus serine show reduced dTMP levels (Fig. 4g). Based on the *tyms-1* versus *thyA* experiments described above (Fig. 4a–e), we proposed that *C. elegans*’s dTMP pool would be limited by *E. coli*’s ability to provide dTMP. Supporting this, we found that *C. elegans*’s dTMP levels are reduced in the SE-FUdR condition, and that single KO of *E. coli*’s *lpd* suppresses this reduction (Fig. 4g). Altogether, the data demonstrate that *E. coli*-mediated conversion of serine/glycine into 5,10-mTHF promotes a reduction of the dTMP pool in *E. coli*, and consequently in *C. elegans*.

We next reasoned that if reduced dTMP availability in the *C. elegans* diet is the main SE-FUdR toxicity mechanism, and not a mere correlation, dietary supplementation with dTMP should suppress SE-FUdR toxicity. To test this prediction, we used the complex experimental setup depicted in Fig. 4h, and described in detail in Supplementary Note 3. A key aspect of this experimental setup is that 5′-fluoroorotic acid (5-FO), a source of FUMP that does not need Upp/Udk-mediated conversion, is used to sensitize *C. elegans* to SE-FUdR toxicity. The first important observation we made is that serine enhances fluoropyrimidine toxicity in a *upp;udk* double KO background (Fig. 4i, j). This observation is consistent with the notion that FUMP is important to sensitize to SE-FUdR toxicity but that increased flux through the pyrimidine salvage pathway is not how serine enhances toxicity (Fig. 2h). Most significantly, dTMP supplementation suppresses SE-FUdR toxicity (Fig. 4i, j, additional control images in Supplementary Fig. 7b). Therefore, the LC–MS data demonstrate that dietary serine inhibits the production of dTMP in bacteria and that in turn reduces the dTMP pool in *C. elegans*, and the dTMP-rescue data demonstrate that scarce dietary thymidine is a major contributor to death in *C. elegans*.

To go one step further and test whether precursors for the synthesis of 5,10-mTHF may limit thymidine-depletion in our experimental setup, we exposed worms to a combination of: (1) 5-FO as the source of sublethal levels of FUMP, (2) 2.5 µg/mL FdUMP, and (3) *deoA E. coli* mutant as the microbe. In this condition, high levels of FdUMP can accumulate because we provide ~10 fold more FdUMP (2.5 µg/mL) than the amount of FUdR we normally use to characterize SE-FUdR (0.25 µg/mL FUdR), and because the KO of *deoA* prevents the conversion of FUdR into 5-FU or FUMP. Nevertheless, despite the expected increase in FdUMP levels, we see no toxicity in *C. elegans* (Supplementary Fig. 7c). However, supplementing these plates with as little as 150 µg/mL of serine leads to >70% lethality, and from there the severity of the toxicity correlates with the amount of serine added to the system (Supplementary Fig. 7c).

Altogether we propose a model in which dietary serine enhances FUdR toxicity through promoting the synthesis of 5,10-mTHF, and with that the formation of the TS inhibitory complex, which results in reduced dTMP production in *E. coli* and thymidine-less death in worms (Fig. 4k). Microbe-mediated thymidine starvation in *C. elegans* can be triggered genetically via KO of *E. coli thyA* or dietarily via supplementation of serine, and likely glycine, in combination with FUdR or FdUMP. Although SE-FUdR toxicity does not act through enhancing the known FUdR-to-FUMP toxicity pathway, it does require FUMP to sensitize the worm to thymidine-less death. This is in line with a previous study demonstrating that nucleotide imbalance in the microbe alone is insufficient to promote toxicity in *C. elegans*^16^. Together, the data show the critical role that four-way interactions can have in fluoropyrimidine toxicity in the host. The results also highlight the need to control animal husbandry conditions to make generalized conclusions about the role microbe and host pathways have in the response to drugs.

### The host distinctively responds to Lth-FUdR and SE-FUdR toxicity

**Fig. 5:**
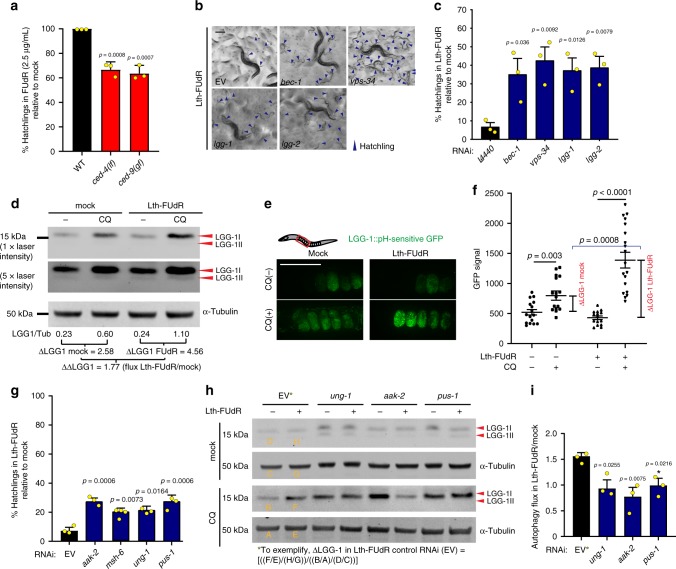
Lth-FUdR activates autophagic cell death in *C. elegans*. Throughout this figure: % hatchlings was analyzed as described in Fig. 1. Statistical significance was assessed via two-tailed unpaired nonparametric *t*-test for % hatchlings quantification. Statistical significance for western blotting ratio was assessed via one-tailed ratio *t*-test. Data are presented as mean values ± SEM, *n* = # independent biological replicates. Source data are provided as a Source Data file. **a** Quantification of progeny viability of WT, *ced-4(n1162)*, and *ced-9(n1950)* mutant *C. elegans* exposed to 2.5 μg/mL FUdR (lower dose of FUdR used to enable detection of enhancers). *n* = 3. **b** Representative images of progeny viability of *C. elegans* exposed to Lth-FUdR while cultured on EORB1 EV or autophagy RNAi clones. Scale bar = 200 µm. **c** % hatchling quantification of treatments represented in **b**. *n* = 3. **d** Representative αLGG-1 western blotting of worms cultured on EORB1 lawn ±Lth-FUdR (7.5 μg/mL) ±8 h exposure to 20 mM chloroquine (lysosomal inhibitor). Two different exposures of αLGG-1 blot are depicted. Autophagy flux estimation and data interpretation described in main text and Methods. *n* = 10. **e** In vivo imaging of embryos expressing LGG-1::GFP(pH-sensitive) treated with ±Lth-FUdR (7.5 μg/mL) and ±8 h of 20 mM chloroquine. Scale bar = 100 μm. **f** Quantification of GFP signal of treatments represented in **e**, two-tailed, unpaired, nonparametric *t*-test. LGG-1::GFP data acquisition, analyses, and interpretation described in main text and Methods. Unpaired nonparametric one-tailed *t*-test was used to singly compare average GFP signal in (+)CQ to (−)CQ (denoted with black brackets and asterisks), and one-tailed ratio *t*-test was used to compare ∆LGG-1 ratios (denoted with blue brackets and asterisks). *n* = 3. **g** Quantification of progeny viability of *C. elegans* exposed to Lth-FUdR (7.5 μg/mL) while cultured on EORB1 EV or RNAi against *aak-2*, *msh-6*, *ung-1*, or *pus-1*. *n* = 3. **h** Representative αLGG-1 western blotting of worms cultured on EORB1 EV or RNAi against *ung-1*, *aak-2* or *pus-1* ± Lth-FUdR (7.5 μg/mL) ±8 h of 20 mM chloroquine. Approach, data analyses, and interpretation described in main text and Methods. **i** Autophagy flux quantification as depicted in **h**, and described in Methods. *n* = 3.

Although the phenotypic outcomes of treatment with Lth-FUdR and SE-FUdR are similar, namely embryonic lethality at low doses and developmental delay at higher doses, the microbial mechanisms leading to these outcomes are distinct. Thus, we next sought to investigate whether the host response to Lth-FUdR and SE-FUdR at a sub-phenotypic level might also be distinct. We first tested whether apoptosis contributes to Lth-FUdR toxicity in *C. elegans*. We found that loss-of-function mutation of the apoptosis activator *ced-4(n1162)* and gain-of-function mutation of the apoptosis inhibitor *ced-9(n1950)* enhance toxicity (Fig. 5a), arguing against apoptosis mediating Lth-FUdR toxicity in *C. elegans*.

Having used a *C. elegans-*mutant approach to determine that apoptotic mechanisms do not mediate Lth-FUdR toxicity in *C. elegans*, we moved to a targeted RNAi screening approach to identify host pathways mediating Lth-FUdR and SE-FUdR toxicity. We performed three- and four-way *C. elegans* RNAi screens of an RNAi sublibrary composed of 361 *C. elegans* genes two steps away from pyrimidine, purine, and serine uptake, synthesis, metabolism, or secretion, built based on a reconciled model of *C. elegans* metabolism we are currently refining (Joshi et al., unpublished). We further added 26 DNA repair, autophagy, and detox pathway genes previously reported to modulate the toxicity of fluoropyrimidines or related compounds^14,22^ (gene list in Supplementary Data 1). To perform RNAi screening using the HB101 background, we developed and validated an RNAi-competent derivative of HB101 that we named EORB1 (Supplementary Fig. 8). Using EORB1, we screened the 387-gene RNAi sublibrary in five conditions: (1) no additives, (2) Lth-FUdR, (3) serine, (4) subLth-FUdR, and (5) SE-FUdR.

We will first describe the results and characterization of the hits of the three-way Lth-FUdR *C. elegans* RNAi screen. SenGupta et al.^22^ and Scott et al.^14^ demonstrated that 5-FU activates autophagy in *C. elegans*, and that death requires the autophagy-related genes *bec-1* (*C. elegans* ortholog of BECLIN 1) and *atg-7* (E1-like enzyme involved in conjugation of the ubiquitin-like proteins LGG-1 and ATG-12 to autophagic membranes)^14,22^. In accordance with these reports, our three-way RNAi screen identified four autophagy genes as suppressors of Lth-FUdR toxicity in *C. elegans* (Supplementary Table 3 and Fig. 5b, c). To better define the role of autophagy, we used 3 approaches to assess the levels of autophagy in worms treated with Lth-FUdR. First, we assessed transcriptional levels of autophagy genes whose expression correlates well with levels of autophagic flux in *C. elegans*^23^, and found increased expression of *atg-16.2*, *atg-18*, and *bec-1* in worms treated with Lth-FUdR (Supplementary Fig. 9a). Second, we assessed autophagy at the protein level. The most cited approaches to measure autophagy in *C. elegans* are the measurement of the number of LGG-1::GFP punctae in in vivo imaging analyses, and using α-GFP antibodies to measure LGG-1::GFP in western blotting assays^24^. However, in isolation, these approaches could be misleading as LGG-1 is subject to autophagic degradation and thus an increased LGG-1::GFP signal could indicate either increased autophagy initiation (increased flux) or decreased lysosomal turnover (decreased flux). Thus, to better assess autophagic flux in *C. elegans*, we developed and immunopurified antibodies against LGG-1. We validated the antibodies using *lgg-1* RNAi and LGG-1 overexpression worms (Supplementary Fig. 9b). We then measured autophagic flux in worms by exposing them to the relevant treatments ± the lysosomal inhibitor chloroquine (CQ). Because CQ blocks lysosomal turnover, the magnitude of the difference in LGG-1 signal between plus and minus CQ reflects the relative level of autophagic flux in any particular condition and can then be compared between conditions (a.k.a. ∆∆LGG-1; see methods for additional details on calculations). Using this metric, we observed 1.5–2 fold increases in autophagic flux in the Lth-FUdR condition (Fig. 5d). Third, as our toxicity readout is embryonic lethality, we assessed autophagic flux in the embryo. For this, we used a previously reported LGG-1 transgenic line^25^ in combination with CQ. In this reporter strain, LGG-1 is fused to a pH-sensitive GFP. Hence, unless lysosomal acidification is perturbed, the GFP signal corresponds to non-acidic autophagosomes (AP). By contrast, in animals treated with an agent that alkalinizes the lysosome (i.e. CQ), the GFP signal corresponds to AP + autolysosomes (AL); hence, the ratio GFP_CQ(+)_/GFP_CQ(−)_ = ∆LGG-1 for a given treatment or control. The simplest interpretations of this readout follow: (1) basal autophagic flux: whichever ∆LGG-1 is observed in wild-type unperturbed animals; (2) reduced or blocked autophagic flux: ∆LGG-1 is smaller (statistically significant) than ∆LGG-1 in the control; and (3) increased autophagic flux: ∆LGG-1 is larger (statistically significant) than ∆LGG-1 in the control. Using this metric, we found a ∆LGG-1 of ~50% in mock and ~300% in Lth-FUdR (Fig. 5e, f), suggesting Lth-FUdR strongly increases autophagic flux. Altogether, Lth-FUdR promotes high levels of autophagy, and 4 different autophagy genes mediate death in the Lth-FUdR condition. Death not only concurrent, but also dependent on autophagy is the definition of autophagic cell death (ACD). Hence, we propose that worms treated with lethal doses of FUdR are dying through ACD.

In line with ACD mediating Lth-FUdR toxicity in *C. elegans*, we found that RNAi against *aak-2* suppresses toxicity (Fig. 5g). *aak-2* encodes for the catalytic subunit of AMP-activated protein kinase (AMPK), a central energy homeostasis kinase that promotes the activation of autophagy^26^ and has been functionally linked to ACD^27^. AMPK responds to several stresses including DNA damage^28^. Among our RNAi screen hits, we found two DNA repair/damage-related enzymes, MSH-6 and UNG-1 (Fig. 5g). The mismatch-repair enzyme MSH-6 has been shown to mediate 5-FU toxicity in *C. elegans*^14,22^. By contrast, *ung-1* has not been previously shown to mediate fluoropyrimidine toxicity in *C. elegans*. UNG-1 is a DNA repair enzyme that catalyzes the removal of uracil misincorporated in DNA. However, if it enters a futile lesion/repair cycle, as when an excess of FdUTP is available to be incorporated into DNA^29,30^ then it promotes DNA damage. Hence, we hypothesized that UNG-1 and AMPK would be part of an axis that activates lethal levels of autophagy in response to FUdR. In support of this hypothesis, we found that RNAi against *ung-1* and *aak-2* suppresses the activation of autophagy otherwise observed in animals treated with lethal doses of FUdR (Fig. 5h, i). Another RNAi hit, *pus-1* (Fig. 5g), provides additional insight into how Lth-FUdR toxicity would be executed in *C. elegans*. From yeast to mammals pseudouridine synthase (PUS-1) converts uridines present in several RNA classes into pseudouridines^31,32^, and pseudouridylation is required for proper maturation and stability of RNAs^33^. However, when uracil is fluorinated PUS-1 is irreversibly linked to it^34,35^, reducing the pool of functional RNAs and promoting toxicity^36^. We then tested whether PUS-1 dysfunction would also be upstream of ACD. Indeed, we found that *pus-1* RNAi suppresses the hyperactivation of autophagy (Fig. 5h, i). Altogether the data show that UNG-1, AMPK, and PUS-1 are upstream of autophagy in the pathway that promotes death in animals treated with a lethal dose of FUdR. Further, that the suppressors of embryonic lethality also suppress the increased autophagic flux, reinforces the notion that ACD executes death in the Lth-FUdR condition.

**Fig. 6:**
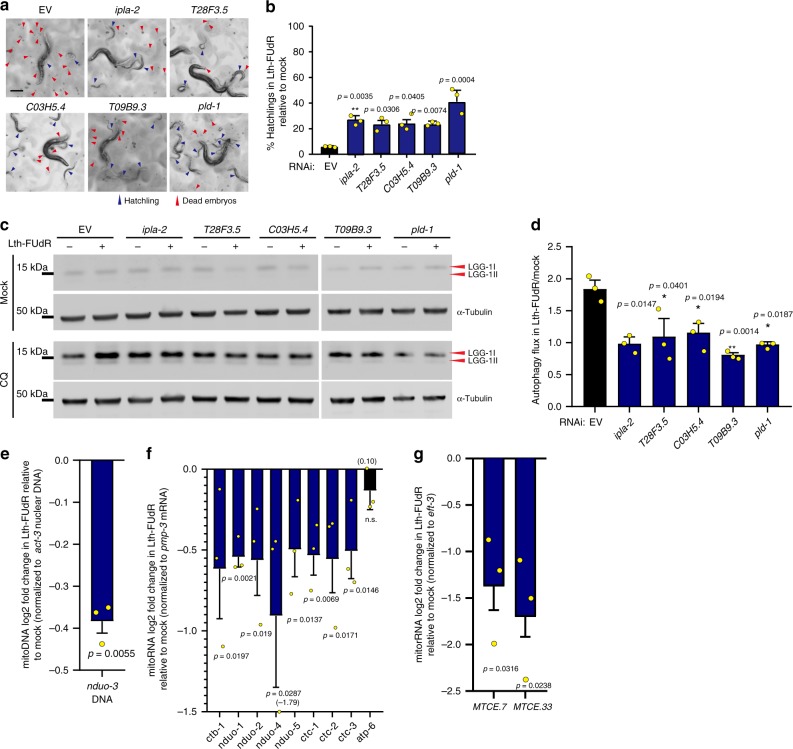
Autophagy activation in Lth-FUdR depends on mitochondrial lipid metabolism. Throughout this figure: % hatchlings was analyzed as described in Fig. 1. Statistical significance for %hatchlings quantification was assessed via two-tailed unpaired nonparametric *t*-test. Statistical significance for western blotting ratio and qPCR fold change was assessed via one-tailed ratio *t*-test. Data are presented as mean values ± SEM, scale bars = 200 µm. *n* = # independent biological replicates. Source data are provided as a Source Data file. **a** Representative images of progeny viability of *C. elegans* exposed to Lth-FUdR (7.5 μg/mL) while cultured on EORB1 EV or RNAi-targeting mitochondrial genes. *n* = 3. **b** Quantification of treatments represented in **a**. *n* = 3. **c** Representative αLGG-1 western blotting analysis of worms exposed to ±Lth-FUdR (7.5 μg/mL) ±8 h of 20 mM chloroquine while cultured on EORB1 EV or RNAi-targeting mitochondrial genes. Data acquisition as described in Methods. **d** Quantification of autophagy flux of treatments represented in **c**. Autophagy flux estimation and interpretation as described in Fig. 5h–I, main text, and Methods. *n* = 3. **e** qPCR analysis of mitochondrial DNA content (*nduo-3*) relative to nuclear DNA (*act-3*) in Lth-FUdR worms relative to mock. *n* = 3. **f** qRT-PCR analysis of the expression/stability of mitochondrially encoded mRNAs relative to the nuclearly encoded mRNA *pmp-3* in worms treated with Lth-FUdR relative to mock. *n* = 3. **g** qRT-PCR analysis of mitochondrially encoded rRNAs normalized to *eft-3* (as previously described) in worms treated with Lth-FUdR relative to mock. *n* = 3.

In addition to being functionally dysregulated by fluoropyrimidines, mammalian UNG-1 and PUS-1 share a mitochondrial subcellular localization^37,38^. This was intriguing because mitochondrial lipids are emerging as key upstream players in non-apoptotic cell death^39–41^, and, in this sense, the Lth-FUdR suppressor *pld-1* is particularly informative because its mammalian homolog, PLD1, produces a lipid signal that activates autophagy^42^. We therefore hypothesized that lipid signals might link mitochondrial dysfunction caused by Lth-FUdR to the activation of lethal levels of autophagy. In support of this hypothesis, we found that *ipla-2*, *T28F3.5*, *C03H5.4*, *T09B9.3*, and *pld-1* not only suppress Lth-FUdR toxicity (Fig. 6a, b) but they also suppress enhanced autophagy (Fig. 6c, d), in line with a model in which lipid signals link mitochondrial dysfunction to ACD.

We then embarked on defining what it is that Lth-FUdR does to the mitochondria. A previous study, found that cytochrome *C* (cytC) abundance is a good predictor of activation of lethal autophagy downstream of loss of mitochondrial membrane integrity^43^. However, in the context of Lth-FUdR, cytC levels do not correlate with toxicity (Supplementary Fig. 9c). This result suggests that loss of mitochondrial membrane integrity is one of several possible insults to the mitochondria that can trigger lethal autophagy, but it is unlikely to be the one triggering it in animals treated with FUdR. In addition, mitochondrial leakage is the most established trigger of apoptosis^44^. Hence, the cytC negative result is in line with apoptosis not being a mediator of Lth-FUdR toxicity (Fig. 5a). We then searched for other insults that may promote the activation of autophagy in animals treated with FUdR. We found no changes in the mitochondrial oxidative stress response as measured by *gst-4* expression (Supplementary Fig. 9d), or the mtUPR response as measured by *hsp-6* mRNA (Supplementary Fig. 9d) and HSP60 protein levels (Supplementary Fig. 9c). However, we did find reduced levels of mitochondrial DNA (Fig. 6e) and mitochondrially encoded mRNAs (Fig. 6f) and rRNA (Fig. 6g) in worms treated with a lethal dose of FUdR. These results align well with PUS-1 and UNG-1 mediating Lth-FUdR toxicity because in mammals futile activation of mitochondrial UNG-1 and malfunction of mitochondrial PUS-1 leads to mitochondrial DNA and RNA toxicity, and mitochondrial dysfunction in vitro and in vivo^38,45^. Therefore, although future studies are warranted to fully dissect the mechanisms executing death in animals treated with lethal doses of FUdR, the data presented here fit a model in which FUdR derivatives (likely FUTP and FdUTP) would be incorporated into the host mitochondrial RNAs and DNA, impairing mitochondrial RNA maturation (via PUS-1 inhibition), and promoting mito DNA damage (via futile UNG-1 activity). In turn, AMPK and lipid signals would transduce mitochondrial damage to the cytosol to activate lethal levels of autophagy.

**Fig. 7:**
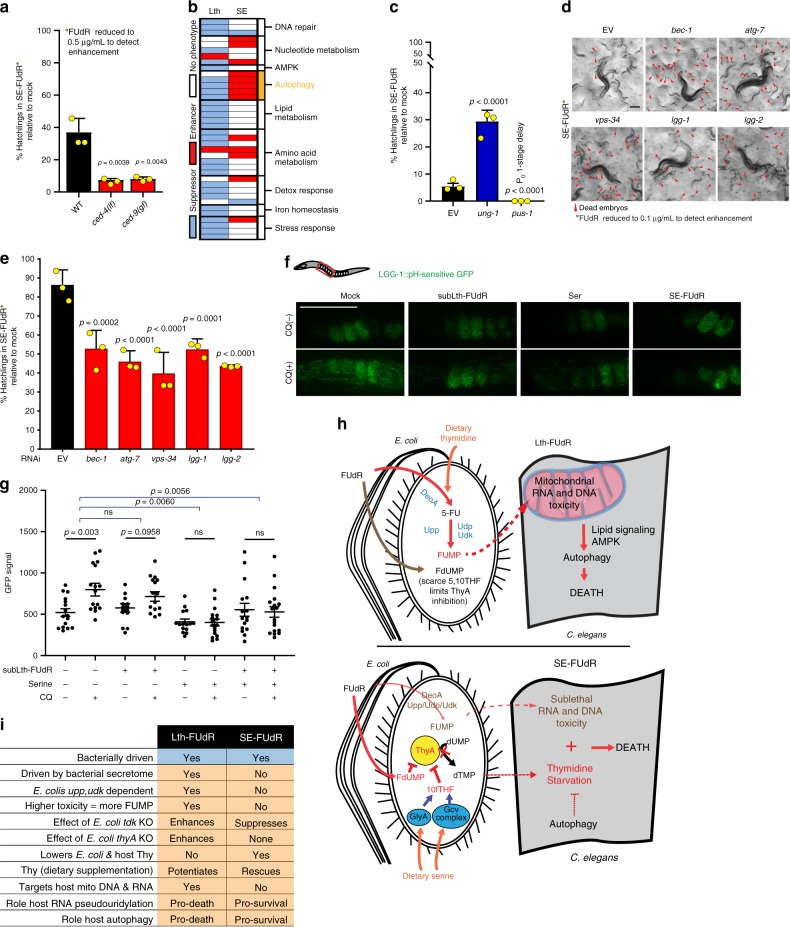
Host response to Lth-FUdR and SE-FUdR are distinct. Throughout this figure: % hatchlings was analyzed as described in Fig. 1. Statistical significance was assessed via two-tailed unpaired nonparametric *t*-test for % hatchlings quantification. Data are presented as mean values ± SEM. *n* = # independent biological replicates. Source data are provided as a Source Data file. **a** Quantification of progeny viability of WT, *ced-4(n1162)*, and *ced-9(n1950)* mutant *C. elegans* cultured on EORB1 lawns treated with 0.5 μg/mL plus 1.5 mg/mL serine (lower dose of FUdR used to enable detection of SE-FUdR enhancers). *n* = 3. **b** GO distribution of the three-way (left) and four-way (right) hits from the *C. elegans* RNAi screen for modulators of Lth-FUdR and SE-FUdR, respectively. Enriched functional class (Fisher’s exact test *p* < 0.005) denoted yellow. **c** Quantification of progeny viability of *C. elegans* exposed to SE-FUdR (1 μg/mL FUdR) while cultured on EORB1 EV or RNAi against *ung-1* or *pus-1*. *n* = 3. **d** Representative images of progeny viability of *C. elegans* cultured on EORB1 EV or autophagy RNAi lawns treated with 0.1 μg/mL plus 1.5 mg/mL serine (lower dose of FUdR used to enable detection of enhancers). Scale bar = 200 µm. *n* = 3. **e** Quantification of treatments represented in **d**. *n* = 3. **f** In vivo imaging of embryos expressing LGG-1::GFP(pH-sensitive) treated in EORB1 lawns with 1 μg/mL FUdR, 1.5 mg/mL serine, and FUdR plus serine, ±6 h on 20 mM chloroquine. Scale bar = 100μm. *n* = 3. **g** Quantification of GFP signal of treatments represented in **f**. Analysis as described in Fig. 5f. **h** Working model of the host response to Lth-FUdR and SE-FUdR. In the Lth-FUdR condition, some derivatives of FUMP generated in the worm (e.g. FUTP) misincorporate into mitochondrial RNAs preventing RNA maturation and function, whereas others (e.g. FdUTP) are incorporated into DNA, promoting detrimental levels of DNA repair. Then lipid signals and AMPK link the consequent mitochondrial dysfunction to the activation of autophagic cell death. In the SE-FUdR condition, mito RNAs and DNA are not major targets. Instead, *C. elegans* die of dTMP deficiency, and its consequent thymidine-less death, which autophagy can alleviate. **i** List of distinctive characteristics of Lth-FUdR and SE-FUdR.

Now, we will describe the results and characterization of the hits of the four-way SE-FUdR *C. elegans* RNAi screen. As the major toxicity mechanism in the SE-FUdR condition is the classic inhibition of TS, and thymidine-less death has been linked to apoptosis, we first tested whether apoptosis was contributing to SE-FUdR toxicity in *C. elegans*. However, we found the apoptosis mutants *ced-4(n1162)* and *ced-9(n1950)* to further enhance SE-FUdR toxicity (Fig. 7a), arguing against apoptosis mediating toxicity in this condition. We then moved onto perform four-way RNAi screening for *C. elegans* genes mediating SE-FUdR toxicity. The screen hits revealed that the host response to SE-FUdR is remarkably distinct from the response to Lth-FUdR. From the nine genes that were hits in both screens, only two show the same phenotype in both conditions (Fig. 7b and Supplementary Table 3). One of these genes is *ung-1*, which suppresses Lth-FUdR (Fig. 5g) and SE-FUdR (Fig. 7c) toxicity. In contrast, the other seven genes that are hits in both screens show opposite phenotypes. *pus-1* suppresses Lth-FUdR toxicity (Fig. 5g) and enhances SE-FUdR toxicity (Fig. 7c), which is in line with a more prevalent role for RNA toxicity in the Lth-FUdR than in the SE-FUdR condition. Most striking, the autophagy genes, as a class, have opposite phenotypes in the two screens. While autophagy mediates Lth-FUdR toxicity, RNAi against the autophagy genes *bec-1*, *atg-7*, *lgg-1*, *lgg-2*, and *vps-34* further enhances SE-FUdR toxicity (Fig. 7d, e), suggesting that autophagy promotes death downstream of fluororibonucleotide toxicity, but protects from death during thymidine starvation. One autophagy gene, *atg-7*, acts distinctively as its inactivation does not suppress Lth-FUdR toxicity but enhances SE-FUdR toxicity. However, autophagy independent from ATG-7 (a.k.a. non-conventional autophagy) has been reported^46,47^, and ATG-7 modulates the DNA damage-responsive tumor suppressor and cell-death mediator p53^48^. Therefore, the protective role of ATG-7 in SE-FUdR toxicity may occur through mechanisms distinct from autophagy. We then measured the levels of autophagy in the SE-FUdR toxicity condition. We found no changes in the levels of expression of autophagy genes (Supplementary Fig. 9e) or autophagic flux by western blots of gravid adults (Supplementary Fig. 9f, g). However, when exposed to serine, embryos show similar GFP signal in the absence and presence of CQ (Fig. 7f, g), suggestive of reduced autophagic flux. Altogether, although several aspects of the death mechanisms remain to be elucidated, it is clear that Lth-FUdR and SE-FUdR are distinctively executed in the host. Further supporting this notion, we observe no changes in mitochondrial DNA (Supplementary Fig. 9h) or RNA content (Supplementary Fig. 9i) in the SE-FUdR condition, and, correspondingly, AMPK and the lipid metabolism genes that suppress Lth-FUdR toxicity do not suppress SE-FUdR toxicity (Fig. 7b). Altogether, the results show that dietary serine not only changes metabolic flux in *E. coli*, and with that the level of toxicity of FUdR, but also redefines the host response to FUdR toxicity (working model in Fig. 7h).

## Discussion

Diet and microbiota are attractive targets for therapeutic intervention. However, the dominance of correlative and in vitro studies on the effects that diet and microbiota have on the host response to drugs has limited the development of therapeutic interventions targeting diet, microbiota, or both. Here, we used a tractable system that enables molecular dissection of four-way diet, drug, microbe, and host interactions in vivo. With this four-way model system, we dissected the microbe and host response to FUdR, and how they both change when serine is supplemented to the diet.

We first show that Lth-FUdR toxicity is bacterially driven. Although thymidine-less death is the best characterized mechanism of FUdR toxicity, *E. coli*-mediated thymidine starvation is not how *E. coli* promotes Lth-FUdR toxicity in our experimental setup. However, when serine is supplemented to the diet, this changes. Dietary serine enables the inhibition of *E. coli*’s thymidylate synthase (TS), reducing the dTMP pool in *E. coli* and consequently in *C. elegans*. Together, the data presented here suggest that the precursors for the synthesis of 5,10-mTHF, an essential TS cofactor, are limiting in our experimental setup. More importantly, the data show that single dietary changes can shift the microbe’s metabolism and, consequently, the host response to a drug to the point of transforming a non-lethal dose into a lethal one. In practical terms, the fact that supplementation with a single dietary metabolite can greatly enhance the potency of *E. coli*-mediated FUdR toxicity as well as shift its mechanism of action emphasizes the need to employ standardized media conditions when studying drug mechanisms in model organisms. Indeed, in *C. elegans* research, peptone concentrations from different commercial providers are not standardized. Thus, some results garnered from *C. elegans* studies using drugs supplemented to the media may be influenced by the varied nutrient compositions of media and the consequent distinct interactions with microbial and host metabolism. More broadly, the mechanisms of action of dietary thymidine and serine show that the microbiota can affect the efficacy or toxicity of drugs through at least two mechanisms: (1) Directly, via metabolizing the drug (i.e. increased conversion of FUdR into FUMP; thymidine mechanism). This mechanism has been exploited to uncover microbiota-drug interactions^49^; and (2) Indirectly, via converting dietary nutrients into metabolites that in turn change the microbe’s capabilities to alter the host response to the drug (i.e. *E. coli*-mediated conversion of dietary serine into 5,10-mTHF enabling thymidine-less death in *E. coli* and hence in the worm; serine mechanism). To the best of our knowledge, this mechanism is demonstrated here for the first time.

The significance of using simplified tractable models of microbe–host co-metabolism resides in unveiling the complexity of the molecular interactions that may affect drug treatment outcomes, and serving as guide for mechanistic studies in higher organisms. At first sight, *C. elegans* may seem too unique to inform host–microbiota interactions in higher organisms. Bacteria serve as microbiota and food source in *C. elegans*^50^. Hence, bacteria are the principal source of micro and macronutrients, and this may seem different from mammals. However, the mammalian gut microbiota has a critical role in providing essential nutrients and in digesting the complex carbohydrates, proteins, and fats that reach the lower gastrointestinal tract in mammals^51^. Furthermore, bacterial lysis, and the consequent release of cell content, is part of the normal mammalian gut dynamics^52^. The enterohepatic system permits exchange of metabolites, byproducts, and xenobiotics between the intraluminal intestine, the bloodstream, and animal tissues^53^. Indeed, microbiota-derived nucleosides and nucleotides can be found in blood and other host organs in mammals^54–56^ including humans^57^, suggesting that microbial nucleotide metabolism could impact host nucleotide metabolism and hence the metabolism of chemotherapeutics beyond *C. elegans*. Similarly, studies in mammals show bacterially converted dietary folates, and bacterially derived serine in host tissues^9,10,12^. It is also notable that panels of probiotic bacteria that include *E. coli* can differentially activate chemotherapeutics in vitro^58,59^. Therefore, although only suggestive, the current mammalian evidence is in line with diet being capable of modulating fluoropyrimidine efficacy and toxicity through altering the metabolism of gut microbes in the clinical setting. Therefore, microbes could account, at least in part, for the variability in fluoropyrimidine responsiveness that cannot be explained by the genetics of the patient or the tumor^60^. Most important, and exemplifying the value of simplified model systems, the notion introduced here that microbe-derived “natural” metabolites can have a significant impact on the efficacy and toxicity of drugs is relevant on its own, because even the most detailed studies to date base the screens for microbial activities modulating drug efficacy or toxicity on biochemical searches for microbe-derived drug derivatives (degradation products or modified versions of the administered drug)^49^. Our work reveals a limitation of these drug-derivative screens, as they would miss microbiota activities (i.e. conversion of dietary serine into 5,10-mTHF) capable of, for example, transforming a non-lethal dose of FUdR into a lethal one.

Notwithstanding, the most surprising finding from this work is that dietary serine also alters, and in cases reverses, the role that host pathways have in the response to FUdR. Examples include RNA modification (*pus-1*) and autophagy (*bec-1*, *lgg-1, lgg-2*, and *vps-34*) executing death in the Lth-FUdR condition and protecting from death in the SE-FUdR condition. Furthermore, even for genes having similar roles in both conditions, the underlying mechanisms may be distinct. For instance, *ung-1* is the only gene with a suppressor phenotype in both conditions. Nevertheless, based on the mitochondrial DNA results, it is likely that UNG-1’s toxic role is due to a futile cycle of removal and reincorporation of fluorouracil in the mitochondrial DNA of FUdR-treated animals. However, in the SE-FUdR condition, mitochondrial DNA is not depleted and fluororibonucleotide toxicity is not the main mechanism of *E. coli*-driven toxicity. Instead, the combination of low levels of dTTP and relative high levels of dUTP and FdUTP would favor the incorporation of fluorinated and non-fluorinated uracils in genomic DNA as previously reported^30,61^. Hence, it is likely that in the SE-FUdR condition, UNG-1 is toxic because it enters a futile cycle of removal and reincorporation of uracil into the genomic DNA. Altogether, even when a surface-level interpretation of the outcome (100% embryonic lethality) would lead one to believe that the same mechanisms underlie death in these two conditions—dead embryos look grossly identical, worms and bacteria are isogenic, and the drug is the same—the underlying mechanisms in the microbe and the host in the presence or absence of dietary supplementation with serine are distinct to the point that the same molecular players have opposite roles (Fig. 7i). Although our study identifies these striking sub-phenotypic distinctions, it leaves many questions unanswered. Future studies would be necessary to fully dissect the underlying death mechanisms in both the Lth-FUdR and SE-FUdR conditions. This will likely be a challenging endeavor, as the mechanisms by which cells die of thymidine-less death have remained unknown for decades^61,62^. Nevertheless, the understanding that distinct mechanisms can underlie the same treatment outcomes should guide future research; in particular, it should encourage limiting the use of correlative studies for translational purposes.

Humans host more than 1500 species in the gut, and the composition varies between and within individuals^63^. Each of these microbes can distinctly metabolize dietary components and drugs. The dietary nutrient and drug derivatives from each microbe can be further metabolized or alter the physiology of other microbes and the host, building chains of events alternatively or simultaneously triggered by dietary, drug, microbe and host metabolites, byproducts, and signaling molecules. Hence, we can speculate that the complexity of drug–microbe–host co-metabolism in vivo is astronomical. Therefore, the complexity of the simplified four-way interactions presented here highlight both the extensive need for mechanistic studies, and the challenges we face to realize the full therapeutic potential of the microbiota.

## Methods

### *C. elegans* and *E. coli* strains

*C. elegans* strains N2 (Bristol, UK), MT2547 (*ced-4* mutant n1162) and MT4770 (*ced-9* mutant n1950) were obtained from the Caenorhabditis Genetics Center (CGC). MAH215^25^ is a kind gift from Dr. Malene Hansen. Unless otherwise noted, experiments were initiated with synchronized L1 larvae obtained by egg bleaching and overnight synchronization in S-buffer. Wild-type *E. coli* strain BW25113 and Keio KO strains were obtained from the *E. coli* Genetic Stock Center. *E. coli* strain HB101 was obtained from CGC. EORB1 and the EORB1 RNAi library were constructed in our lab.

### *E. coli* culturing and compound supplementation

For every biological replicate fresh *E. coli* streaks or library stamps on LB-carbenicilin 50 µg/mL (RNAi clones) or LB-kanamycin 25 µg/mL (Keio KO library) were used. Bacterial cultures were started from single colonies or using a sterilized inoculating hedgehog, and grown overnight for 14–16 h. Keio clones were grown overnight in LB-kanamycin 15 µg/mL, RNAi clones were grown overnight in LB-carbenicilin 50 µg/mL in the absence of IPTG (or any other additives). The parental strain BW25113 was grown in plain LB. For aeration, flasks were shaken at 250 rpm, and 1.2 mL deep 96-well plates at 1000 rpm. For targeted experiments, bacteria were harvested by centrifugation at room temperature and resuspended to OD_600nm_ = 20 in S-buffer (~20× concentrated). For screening, 1.2 mL bacterial cultures were resuspended with 20 µL of S-buffer. Concentrated *E. coli* were seeded onto NGM or NGM-RNAi plates immediately and never exposed to the cold.

For dietary supplementation, metabolites were dissolved in water (unless otherwise stated), filter-sterilized, seeded on NGM or RNAi plates, and dried in biosafety hood. Concentrated bacteria were seeded as soon as metabolites dried out. FUdR was dissolved in water to 100× concentration, filter-sterilized, and added directly onto bacterial lawns immediately after lawns were dried. Seeding dietary supplement, fresh bacteria, and FUdR in that order, and adding supplements and FUdR within a 2 h window of seeding fresh bacteria is critical to observe the full effect of the supplements. Synchronized hatchlings were seeded the same day for all experiments except for *C. elegans* RNAi experiments (24 h later to activate RNAi). When post-developmental transfer (i.e. embryogenic competence in Suppl. Figure 1e) was necessary, worms were grown in the *E. coli* background in which they were later tested.

### Imaging and image analysis

Percent hatchling was measured by taking ≥5 images of each treatment or mock plate per biological replicate, and at least three independent biological replicates were carried out for all assays. Images were taken on Zeiss Axio Zoom.v16 dissecting microscope, PlanNeoFluar Z ×2.3/0.57 FWD objective, zoom ×30. Hatchlings, live and dead eggs and adults were quantitated assisted by ImageJ object counting tool. Values in figures are presented as “% hatchling relative to mock”, meaning the number of hatchings was first normalized to total progeny (hatchlings + live embryos + dead embryos) in each treatment and then normalized to the % hatchlings in the corresponding non-FUdR (mock) treatment. This provides a quantitative measurement controlling for other variables such as the time of scoring. For Keio clones and RNAi experiments, treatments are normalized first to mock of the same Keio clone or RNAi and then to WT, which takes into account the potential effect of the Keio or RNAi clones on worm health. However, we did not observe Keio or RNAi only effects in any of the *E. coli* or worm genes inactivations reported as hits.

Estimation example: if BW25113 + 1 µg/mL FUdR = 21 hatchlings/185 progenies (hatchlings + live embryos + dead embryos), and BW25113 untreated = 197 hatchlings/201 progenies, this implies % hatchlings for BW25113 in FUdR relative to untreated is 11.58% (11.35/98 × 100). Then, if *deoA* shows hatchling/progeny ratios of 168/176 in FUdR and 194/199 in control, by the same calculation *deoA* % hatchling is 97.91%; thus, if reproducible, *deoA* is a suppressor.

For enhancers, lower doses of FUdR are used and the calculations take into account the effects of FUdR relative to wild type, but in this case the WT + FUdR will show subtle toxicity. As an example, if BW25113 + 0.5 µg/mL FUdR = 80 hatchlings/152 progenies and BW25113 untreated = 198 hatchlings/200 progenies, % hatchlings for WT BW25113 in this condition is 53.16%. If *ndk* + 0.5 µg/mL FUdR has 25 hatchlings/148 progenies and *ndk* untreated has 205 hatchlings/208 progenies, % hatchlings in worms fed *ndk* is 17.13%, so *ndk* is an enhancer because when cultured on this *E. coli* mutant worms produce less viable progeny than when fed wild-type *E. coli*.

All toxicity measurements were repeated ≥ 3 times and the mean ± SEM are presented.

Occasionally, embryogenic competence was calculated to identify enhancers of toxicity. The calculation is inclusive of hatchlings, live eggs and dead eggs produced per worm, and is influenced by the effect of FUdR (±supplements) on both the rate of development and the fertility of the P_0_s. Therefore, lesser embryogenic competence or P_0_ developmental delay compared to wild-type or unsupplemented reveals enhancers, whereas increased embryogenic competence reveals suppressors. Specifically, 10 worms were singly transferred to test plates, and allowed to lay progeny for 24 h. Next day the total number of progeny (live + dead embryos) were counted per plate. Normalization of embryogenic competence was done as described above for %hatchlings.

### Dietary metabolite four-way screen

Amino acids were freshly dissolved to 10 mg/mL in water (except tyrosine: 1 mg/mL), aluminum foiled, rocked for 12 h at RT, and filter-sterilized. Seven 1:2 serial dilutions were made and amino acids were seeded to the appropriate concentrations into 96-well plates with 100 μL NGM per well. Once dry, wells were seeded with 8 µL of fresh 20× HB101. Once dry, 2 plates (duplicate) were seeded with 5 μL of 250 µg/mL FUdR (final 12.5 µg/mL). The remaining two plates (duplicate) were left as no-FUdR controls to test the potential toxicity of the amino acids. Once dry, 25 synchronized hatchlings were seeded per well and incubated at 20 °C. Altogether, the following conditions were tested: (1) Negative control: 12.5 µg/mL FUdR-only, which leads to 100% sterile adults but no developmental delay; (2) Positive control: 12.5 µg/mL FUdR supplemented with 5 mg/mL thymidine, which leads to 100% larval arrest; (3) Amino acid toxicity control: wells supplemented only with the 8 doses of amino acids (but no-FUdR), to test for the potential toxicity of the amino acids; and (4) Screening wells: wells supplemented with the 8 doses of amino acids and 12.5 µg/mL FUdR. After 60 and 72 h, wells were scored as follows for worm developmental stages: 1 = L1/dead, 2 = L2 larvae, 3 = L3 larvae, 4 = L4 larvae, 5 = Young adults (<5 eggs in body), 6 = Gravid adults (>5 eggs in body). Only wells which showed a ≥1 stage delay in FUdR + amino acid compared to FUdR-only, and the AA showed no toxicity on its own, were considered hits.

### Supernatant and pellet test

Saturated overnight *E. coli* cultures were re-inoculated 1:50 in liquid NGM (Nematode Growth Media without agar), and grown to OD_600nm_ ~1, at which point water (mock), Lth-FUdR (50 µg/mL), or subLth-FUdR (1 µg/mL) ± thymidine (5 mg/mL) or serine (1.5 mg/mL) were added. After 2 more hours of incubation at 37 °C, bacteria were pelleted and resuspended in ≥50 volumes of water three times to remove residual FUdR from the bacterial suspension. On the final wash, OD_600nm_ absorbance was measured and bacteria were resuspended to OD_600nm_ = 20, and 100 µL were seeded onto NGM plates. Once lawns were dried, 100 hatchlings were seeded and incubated at 25 °C. At multiple times between 60 and 96 h plates were imaged for scoring of developmental stages and fertility.

The same setup was used for supernatant experiments, but after centrifugation the supernatants were taken on ice, filtered-sterilized through a 0.22-μm filter, lyophilized overnight, and resuspend in water to make 1 and 5× concentrated supernatants. 100 µL of the sterile supernatant resuspensions were seeded on top of *upp;udk;udp* triple KO *E. coli* lawns. We used 3KO lawns to avoid in-plate conversion of FUdR remaining in the supernatant. Once supernatants dried, 100 hatchlings were seeded and incubated at 25 °C. At multiple times between 60 and 96 h, plates were imaged for scoring of developmental stages and fertility.

### *E. coli* four-way screen

Keio screen for mediators of SE-FUdR toxicity was performed at 25°C in 8 conditions: (1) mock (water), (2) FUdR 0.25 µg/mL, (3) serine 1.5 mg/mL, and (4–8) serine 1.5 mg/mL plus FUdR from 0.05 to 0.25 µg/mL (setup depicted in Fig. 3a). Keio clones were grown overnight in 1.2 mL of LB-kanamycin in deep 96-well plates at 37 °C and 1000 rpm. Cultures were pelleted, supernatants discarded, and pellets resuspended in 20 µL of S-buffer. Eight microliters of bacterial suspension were seeded into wells containing 100 μL of NGM plus or minus serine. Once bacteria dried, 5 μL of 1, 2, 3, 4, or 5 µg/mL FUdR were seeded onto bacteria lawns (final doses 0.05, 0.1, 0.15, 0.2, and 0.25 µg/mL, respectively). Once dried, 50 synchronized hatchlings were seeded and incubated at 25 °C. The non-FUdR controls allowed us to determine whether any given *E. coli* KO clone would adversely affect development or fertility on their own or in combination with serine only. FUdR-only wells were scored after 60–72 h relative to WT *E. coli* BW25113 as follows: −2 = no hatchlings, −1 = fewer hatchlings, 0 = similar to WT control, 1 = more hatchlings than control, 2 = similar to no-FUdR control. FUdR + serine wells were scored after 60–96 h for developmental delay or embryogenic competence relative to WT *E. coli* BW25113 control as follows: −2 = severely delayed P0, −1 = moderate delay P0/few eggs laid, 0 = similar to control (sterile adult), 1 = some hatchlings, 2 = similar to no-serine control. Only genes showing a suppressor or enhancer phenotype in the FUdR plus serine condition at multiple doses or in >2 screen repeats were considered hits. All Keio hits were verified by PCR and sequencing. Primary hits are presented in Supplementary Table 2 as: blue = suppressor of toxicity; orange = enhancer of toxicity. Light blue or orange, represents phenotype observed in only 1 of screen 3 repeats. Hits belonging to overrepresented metabolic pathways were retested in 6 cm NGM plates and quantitated for % hatchlings in sublethal FUdR (0.25 µg/mL) ± serine (1.5 mg/mL), and the results are presented in main figures. Primary screen hits that were not retested in 6 cm plates are depicted as NRT in Supplementary Table 2. Retested and verified hits and non-hits are marked as “√”, and retested but not validated primary hits (phenotype did not repeat) are marked as “X”.

### Bacterial growth in plate

*E. coli* BW25113 and HB101 were cultured and seeded in NGM ± additives plates as normally done for Lth-FUdR or SE-FUdR tests. After 48 h exposure to treatments, cells were recovered from the plates, resuspended in equal volumes, and biomass (OD_600nm_) and viability (CFU of serial dilutions from 10^0^ to 10^−7^) were quantitated.

### Bacterial growth in liquid

HB101, BW25113, and Keio hits were hedgehog seeded from frozen stocks onto LB agar omnitray plates. Next day, 100 µL of liquid NGM ± subLth-FUdR ± serine were stamp-seeded in duplicate. Absorbance at 600 nm was recorded longitudinally using a SpectraMax plate reader maintained at 37 °C in a continuous shaking mode. Measurements were independently carried out more than three times.

### Bacteria and *C. elegans* metabolomics

For Lth-FUdR and dietary supplementation-related metabolomics, single colonies of *E. coli* BW25113 or HB101 were used to inoculate 500 mL of LB and incubated overnight for 14 h at 37 °C 250 rpm. Bacteria were harvested by centrifugation, and resuspended in 25 mL of S-buffer. Concentrated bacteria were seeded on nylon membranes placed on the surface of the NGM plates to avoid “contamination” of the bacteria with NGM agar media. For this, 5 mL of concentrated bacterial suspension were dried onto 90 mm Nylon membranes (VWR 7402-009) by vacuum filtration in a sterile porcelain Buchner funnel. The nylon membranes loaded with bacteria were placed on the surface of 15 cm NGM agar plates with or without the respective supplementations (i.e. ±FUdR and ±thymidine or ±serine). After 24 h at 25 °C, *E. coli* were harvested by washing the bacteria off the membrane with 50 mL of cold liquid NGM. Bacteria were then washed two more times with 50 mL of cold water. To confirm effectiveness of the treatments, 100 µL of bacterial suspension were seeded on NGM plates without any additives, hatchlings were seeded on these lawns, and incubated for 60 h at 25 °C; a time at which they were scored for progeny viability. The remaining of the washed bacterial pellets were flash frozen, lyophilized, and kept at −80 °C for later extraction as described below. Samples verified via parallel hatchling-viability controls were processed for LC–MS analyses.

For supernatant analyses of thymidine-enhanced toxicity, single colonies of *E. coli* BW25113 were used to inoculate 20 mL of LB broth and incubated overnight for 14 h at 37 °C 250 rpm. Next morning cultures were pelleted, washed, and resuspended in equal volume of liquid NGM. Ten milliliters of this bacterial resuspension were used to inoculate 500 mL of liquid NGM supplemented with mock, or subLth-FUdR ± thymidine (5 mg/mL), and incubated for another 2 h at 37 °C 250 rpm. Mock and treated cultures were then harvested by centrifugation. The supernatants were filter-sterilized to remove residual bacteria. Aliquots (10 mL) were spiked in with the internal standards listed below, frozen, lyophilized, and reconstituted right before LC–MS in 1:1 acetonitrile: water. The bacterial pellets were washed two more times with 50 mL of cold water, flash frozen and lyophilized, and kept at −80 °C for later extraction as described below.

To generate worm metabolomics samples, 50,000 synchronized 1-day gravid adult worms were harvested, washed in a 40 µm mesh 1× with 50 mL of NGM, incubated for 5 min in clean media to allow gut clearance, and then mesh-washed again with 50 mL cold liquid NGM and 1× with 50 mL of cold water, and immediately frozen in liquid nitrogen and lyophilized, and kept at −80 °C for later extraction as described below.

Right before resuspending the lyophilates, a master mix of internal standards was prepared by mixing the following compounds at a final concentration of 20 ng/µL in HPLC-grade methanol: (1) 1,3-15N2 Uracil (Cambridge Isotope lab NLM-637-PK); (2) Uridine-13C9,15N2 5′-triphosphate (Sigma #645672); and (3) Glycine-13C2, (Sigma #283827). Then, the spike-in control master mix was diluted 1:50 in 80% methanol. 20 mg of lyophilized bacteria or 5 mg of lyophilized worms were resuspended in 500 µL of the diluted internal standard solution. Samples were mixed with 200 µL of 100 µm silica beads and disrupted 5× for 30 s in a mini-beadeater^TM^-8 disruptor with cooling on ice for 2 min after each cycle. Extracts were cleared through two rounds of 15 min centrifugation at 4 °C and 20,000×*g*, and then lyophilized. To measure endogenous metabolites, samples were reconstituted right before LC–MS in 1:1 acetonitrile: water. Samples were separated on a Luna aminopropyl column (3 μm, 150 mm × 1.0 mm I.D., Phenomenex) or a CORTECS T3 column (2.7 µm, 150 mm × 2.1 mm I.D., Waters) and analyzed using an Agilent 6530 Q-TOF, an Agilent 6540 Q-TOF, or a ThermoScientific Q Exactive Plus. The Luna column was used in negative mode with the following buffers and linear gradient: A = 95% water, 5% acetonitrile (ACN), 10 mM ammonium hydroxide, 10 mM ammonium acetate; B = 95% ACN, 5% water; 100% to 0% B from 0 to 30 min and 0% B from 30 to 40 min; flow rate 50 μL/min. The T3 column was used in positive mode with the following buffer and linear gradient: A = 95% water, 5% ACN, 10 mM ammonium acetate, 0.1% formic acid; B = 95% ACN, 5% water; 0% to 100% B from 0 to 30 min and 100% B from 30 to 40 min; flow rate 200 μL/min. The identity of each metabolite was confirmed by comparing retention times to standard compounds and tandem MS data with the METLIN metabolite database.

To measure fluorometabolites, samples were reconstituted right before LC–MS in 2:1:1 water:methanol:acetonitrile, and 3 μL was further analyzed by liquid chromatography-mass spectrometry (LC–MS) as follows. Metabolite profiling was performed using Ultimate 3000 UHPLC (Dionex) coupled to Q Exactive Plus-Mass spectrometer (QE-MS, ThermoScientific). A hydrophilic interaction chromatography method (HILIC) employing an Xbridge amide column (100 × 2.1 mm i.d., 3.5 μm; Waters) was used for polar metabolite separation. The LC method is modified from a previous study^64^: The HPLC (Ultimate 3000 UHPLC) is coupled to QE-MS (ThermoScientific) for metabolite separation and detection. An Xbridge amide column (100 × 2.1 mm i.d., 3.5 μm; Waters) is employed for compound separation at room temperature. The mobile phase A is 5 mM ammonium acetate and in water, pH 6.8, and mobile phase B is acetonitrile. The linear gradient used is as follows: 0 min, 85% B; 1.5 min, 85% B, 5.5 min, 35% B; 10 min, 35% B, 10.5 min, 35% B, 14.5 min, 35% B, 15 min, 85% B, and 20 min, 85% B. The flow rate was 0.15 mL/min from 0 to 10 min and 15 to 20 min and 0.3 mL/min from 10.5 to 14.5 min. The QE-MS is equipped with a HESI probe with related parameters set as below: heater temperature, 120 °C; sheath gas, 30; auxiliary gas, 10; sweep gas, 3; spray voltage, 3.0 kV for the positive mode and 2.5 kV for the negative mode; capillary temperature, 320 °C; S-lens, 55; scan range (*m/z*): 70 to 900 for pos mode (1.31 to 12.5 min) and neg mode (1.31 to 6.6 min) and 100 to 1000 for neg mode (6.61 to 12.5 min); resolution: 70,000; automated gain control (AGC), 3 × 10^6^ ions. Customized mass calibration was performed before data acquisition. LC–MS peak extraction and integration were performed using commercially available software Sieve 2.2 (ThermoScientific).

For all metabolites, peak sizes of the target metabolites (P) were normalized to the corresponding internal standard (IS) peak area (amino acids to Glycine-13C2, nucleotides to UMP-13C9,15N2, and nucleosides to Uracil-15N2). The normalized peak value from the treatments was then normalized to the mock control from the same biological replicate. Therefore, target metabolite relative abundance was estimated as follows (P/IS)_treatment_/(P/IS)_mock_ from 3 to 5 independent biological replicates

### EORB1 strain construction

To construct the RNAi-competent EORB1 strain, *E. coli* HB101 was first transiently made *recA+*. Then *rnc14* was interrupted with mini-Tn10 transposon introduced via P1 transduction. Transduced colonies were selected in 25 µg/mL tetracycline (Tet). Tet-resistant colonies were picked and re-streaked for two rounds, and then cured of the *recA* plasmid by growing in LB at 44 °C. Streaking onto LB plates containing chloramphenicol (25 µg/mL) and treating with UV confirmed loss of chloramphenicol resistance and the reappearance of sensitivity to UV. Loss of RNAseIII function should prevent the maturation of rRNAs. The rnc-phenotype was confirmed as accumulation of the 30S rRNA precursor (Supplementary Fig. 8a). T7 polymerase (under the control of the *Lac*UV5 promoter) and the transcriptional repressor *Lac*I were then introduced via lysogenization (λDE3 Lysogenization Kit; Novagen 69734). The presence of inducible T7 polymerase was confirmed by western blot (Supplementary Fig. 8b). The final EORB1 strain has the genotype [HB101], rnc-, lacI-lacUV5p-T7. EORB1 was confirmed to be competent for feeding RNAi by phenotypic analysis. RNAi against the genes *daf-2*, *unc-22*, *dpy-13*, and *pos-1* displayed the expected phenotypes when delivered via feeding RNAi from EORB1 (Supplementary Fig. 8c–f). To construct the EORB1 library, we miniprepped (QIAprep 96-plus Miniprep Kit; Qiagen 27291) the screened constructs from HT115 Ahringer RNAi library. EORB1 was made chemically competent by CaCl_2_ preparation. 5 μL of the plasmid minipreps were added to 50 µL of chemically competent EORB1 heat shocked at 42 °C, and grown overnight in liquid LB-carbenicilin 50 µg/mL. Transformants underwent a second and third round of selection on solid and liquid LB-carb50, after which glycerol stocks were made.

### Four-way *C. elegans* RNAi screen and verification

RNAi screens were performed at 25 °C in 96-well plates. RNAi clones were grown in 1.2 mL of LB carbenicillin for 12–16 h in 96-deep-well plates at 37 °C and 1000 rpm. Cultures were harvested by centrifugation. Pellets were suspended in residual LB volume, and 8 μL were seeded into wells containing 100 μL of NGM with 50 µg/mL carbenicillin and 5 mM IPTG (though 1 mM IPTG is sufficient). Wells were then seeded with ±2.5 µg/mL FUdR, and ±5 mg/mL thymidine or ±1.5 mg/mL serine as appropriate. After drying in biosafety hood, plates were left overnight at room temperature to allow RNAi induction. The next day, 25 L1 larvae were seeded and grown for 60–72 h. At this point, controls were confirmed to have 0 hatchlings and plates were scored as follows: 0 = similar to control, 1 = ~1–10 hatchlings, 2 = ~11–50 hatchlings, 3 = ~51–100 hatchlings, 4 = >100 hatchlings, and −1= developmental delay. All RNAi hits were sequence verified. Primary hits are presented in Supplementary Table 3 as: blue = suppressor of toxicity; orange= enhancer of toxicity; and white= no different from WT control. Light blue or orange, represents phenotype observed in only 1 of screen 3 repeats. Hits belonging to overrepresented pathways were retested and quantitated for % hatchlings in 6 cm plates, and the results are presented in main figures. Not retested hits are depicted as NRT in Supplementary Table 3, whereas retested and verified hits and non-hits are marked as “√”, and retested RNAi clones in which the phenotype did not repeat are marked as “X”.

### LGG1 antibody

Anti-LGG1 (Rabbit) antibodies were generated by Covalab (Villeurbanne, France) against peptides FEKRRAEGDKIRRKY and GQLYQDHHEEDLFLY (sequence optimized and kindly shared by Vincent Galy). Serum was immunopurified and anti-LGG-1 specificity was validated in western blots using WT, *lgg-1* RNAi, and *lgg-1* OE samples as controls (Supplementary Fig. 9b).

### Western blotting

For all western blots to measure autophagy levels, samples were prepared from two plates of 2000 worms grown at 25 °C for each treatment. After 50 h, 2000 worms from one of plates were harvested and reseeded in NGM plates with the same additives (i.e. ±subLth-FUdR and ±serine) plus 20 mM chloroquine (CQ). After 8 h of treatment, worms were harvested from both sets of plates (±CQ), washed 3X with S-buffer to remove residual bacteria, and immediately frozen in liquid nitrogen. Frozen worm pellets were resuspended in 2 volumes of 1× RIPA buffer (Sigma #R0278) and sonicated. Aliquots of soluble proteins were quantitated using BCA Thermo kit (Pierce 23227), and the rest mixed with 3× SDS-PAGE sample loading buffer, and incubated for 5 min at 85 °C. 30 µg of protein were loaded to each lane of a 4–12% Bis-tris gel (Fisher NP0322BOX), and ran for 55 min at 200 V in MES running buffer. Gels were transferred to 0.2-μm nitrocellulose membranes in transfer buffer (Fisher NP0006) with 20% methanol at 30 V for 45 min. The membranes were stained with Ponceau red to evaluate the quality of the SDS-PAGE and transfer, and then blocked with Intercept^®^ (PBS) Blocking Buffer (Li-Cor 927-70001) with 0.1% Tween for 4 h. Membranes were exposed to primary antibodies including α-LGG1 1:250 (custom made by Covalab), anti-α-tubulin 1:10,000 (DSHB 4A1), α-HSP60 1:1000 (DSHB HSP60), α-cytC 1:1000 (Abcam 37BA11) overnight at 4 °C. Membranes were washed 3 × 15 min with PBST (0.1% Tween) and incubated with secondary antibodies 1:10,000 for 1 h at room temperature (IRDye® 800CW Goat anti-Mouse IgG Secondary Antibody, CAT#925-32210, and IRDye® 800CW Goat anti-Rabbit IgG Secondary Antibody, CAT#925-32211), and imaged by Li-Cor Odyssey imager. For cytC western blots, samples were prepared from a single plate and processed similarly to a previous protocol with the modifications described above^43^.

Autophagy flux in treated animals relative to mock was calculated by measuring integrated density (*I*) of each band and normalized as shown in Fig. 5h. Autophagy flux relative to mock was then estimated as: ∆∆LGG-1 = ((LGG-1_treatment+CQ_ /Tubulin_treatment+CQ_)/(LGG-1_treatment no CQ_ /Tubulin_treatment no CQ_))**/**((LGG-1_mock+CQ_ /Tubulin_mock+CQ_)/(LGG-1_mock no CQ_ /Tubulin_mock no CQ_)).

### In vivo fluorescent reporter-based measurement of autophagic flux

Two hundred 1-day-old adult worms (strain MAH215) from each treatment (mock, sublth-FUdR, serine, sublth-FUdR+serine, or Lth-FUdR) were harvested with S-buffer, washed 3×, and incubated 30 s with egg-prep bleaching solution. Worms were then washed 3× in S-buffer, and mounted onto agar pad on glass slides (Thermo, 3011) with #1.5 coverslip (Fisher 1.5 22 × 22 mm).

*Z*-stacks to measure autophagic flux in embryos were captured on Nikon Eclipse Ti spinning disc confocal microscope, 40×/1.3NA objective, 500 ms exposure time and 80% laser intensity. All the images from each biological replicate were identically processed using ImageJ. First, maximum projections of the fluorescent and bright field channels were created in ImageJ. Then the embryos were cropped out for analysis. ImageJ plot profiling combined with thresholding was used to detect and quantitate LGG-1::GFP dots. Average GFP signal of >15 individuals from ≥4 biological replicates are depicted for each treatment. Then, ∆LGG-1 = (average GFP signal in CQ(+)) / (average GFP signal in CQ(−)) is calculated for each treatment (i.e. Lth-FUdR) and control. Lastly, all repeats (*n* = 4) of ∆LGG-1_treatment_ and ∆LGG-1_control_ are compared using ratio t-test.

### qPCR analysis

One-day gravid adults were harvested, washed in a 40 µm nylon mesh, and quickly frozen in liquid nitrogen. Samples were kept at −80 °C until RNA extraction. Total RNA was isolated using TriReagent (MRC). To quantitate expression of mitochondrially encoded RNAs (no introns), DNA was removed by DNAse I (Sigma AMPD1) treatment prior to retrotranscription with random hexamer primers. All qRT-PCR reactions were performed in triplicate. Median ± SEM of *dd*Ct is reported^65^. For measurement of mitochondria to nuclear DNA ratios, 100 gravid worms from mock and treatments were lysed with Worm Lysis Buffer (50 mM KCl; 10 mM Tris pH 8.3; 2.5 mM MgCl_2_; 0.45% NP-40; 0.45% Tween-20; 0.01% gelatin) with 0.3% proteinase K and the supernatants containing DNA were collected and used as template. iTaq Universal SYBR Green Supermix (Biorad 1725120) reactions run in BioRad CFX96 thermocycler were analyzed using *dd*Ct^65^.

### Statistics and data representation

All statistical analyses were performed in Graphpad Prism. Outliers were detected and removed from analyses using the ROUT method. For %hatchlings, embryogenic competency, and GFP intensity quantifications, unpaired nonparametric *t*-test was used to make single comparisons between a specific treatment and mock control in. Ratio *t*-test was used to compare all ratios including qPCR fold changes, ∆LGG-1, CFU, and normalized LC–MS ratios. Unless otherwise stated, exact *p*-values are provided within the figures. Unless otherwise stated, data in this study are presented as mean values ± SEM. All experiments were performed and quantitated at least three independent times.

### Reporting summary

Further information on research design is available in the Nature Research Reporting Summary linked to this article.

## Supplementary information

Supplementary Information

Description of Additional Supplementary Files

Supplementary Data 1

Reporting Summary

## Data Availability

The source data underlying Figs. 1b–m, 2b–j, 3b, c, 4a–g, 4i, j, 5a–i, 6a–h, 7a–g, Supplementary Figs. 1b–f, 3b, c, 5a–e, 6, 7a–e, 8a–f, 9a–i, and Supplementary Tables 1–3 are provided as a Source Data file. All other data supporting the findings of the study are available from the corresponding authors upon request.

## Source data

Source Data
