# Supplementary material for: Dietary serine-microbiota interaction enhances chemotherapeutic toxicity without altering drug conversion
Source: Nat Commun. 2020 May 22;11:2587. doi: 10.1038/s41467-020-16220-w (PMC7244588; doi:10.1038/s41467-020-16220-w)
Supplement: Supplementary file 1 — Supplementary Information [file 41467_2020_16220_MOESM1_ESM.pdf]

Supplementary Information

Dietary serine-microbiota interaction enhances chemotherapeutic toxicity without altering drug conversion

Ke and Saba, et.al

Supplemental Figure 1

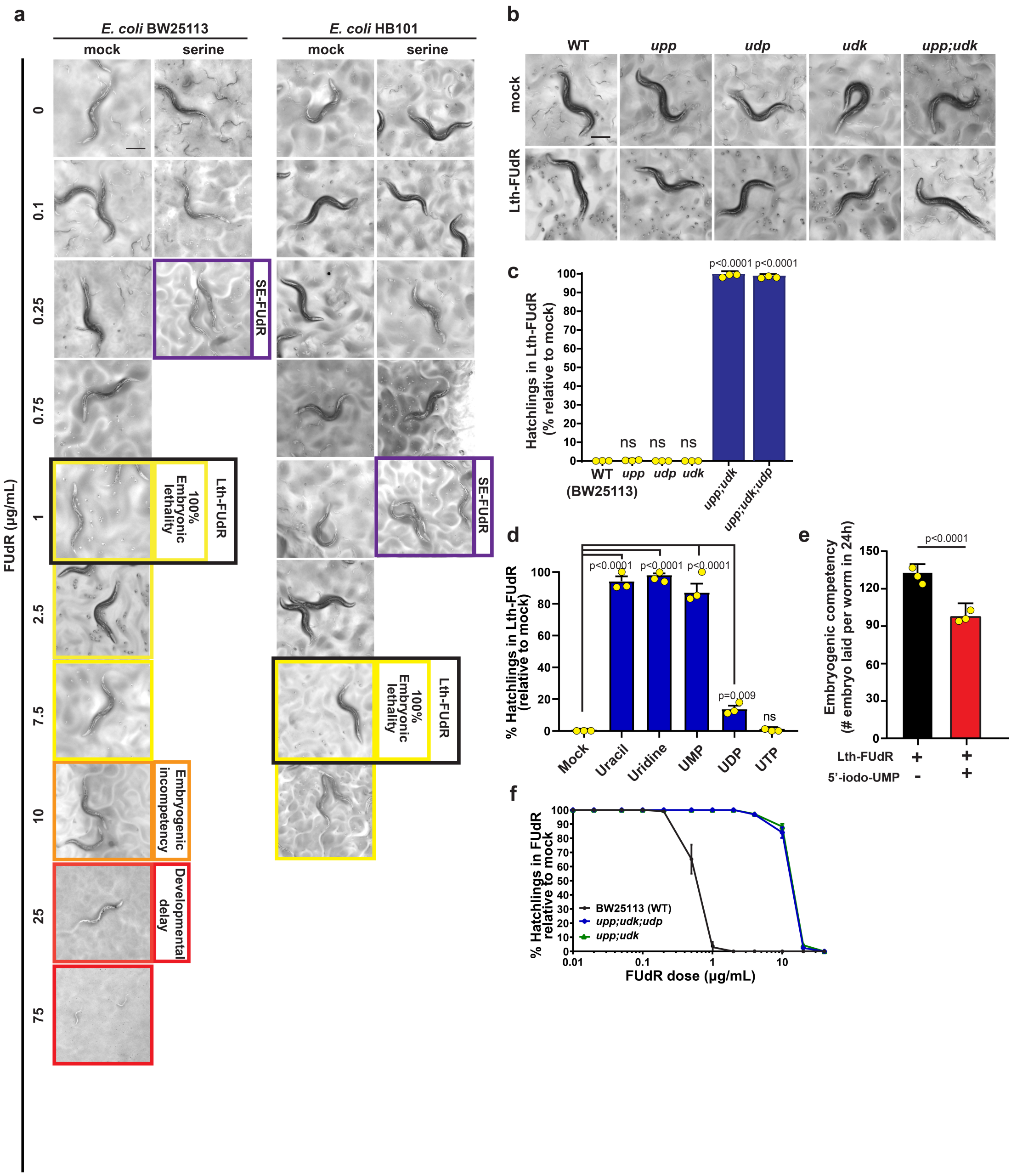

Supplementary Figure 1. FUdR-to-FUMP conversion in *E. coli* drives Lth-FUdR toxicity in *C. elegans.*

Throughout this figure: % hatchlings is estimated as [live hatchlings/(live hatchlings + live embryos + dead embryos)] in condition of interest (i.e. Lth-FudR) relative to % hatchlings in mock of the same *E. coli* or *C. elegans* genotype; ≥5 images of each treatment were quantitated. Statistical significance was assessed via two-tailed unpaired nonparametric t-test for % hatchlings quantification. Data are presented as mean values ± SEM, scale bars = 200μm. n= # independent biological replicates. Source data are provided as a Source Data file.

(**a**) Representative images of the toxic effect of increasing doses of FUdR in the absence or presence of 1.5mg/mL serine on *C. elegans* cultured on *E. coli* BW25113 or HB101. n>10. (**b**) Representative images of progeny viability of *C. elegans* exposed from L1 to mock or Lth-FUdR (1μg/mL) while cultured on WT *E. coli* (BW25113), or the single mutants *upp*, *udp*, and *udk*, or the double mutant *upp;udk*. The WT and *upp;udk* images are also shown for the same conditions in Fig. 1B. (**c**) Quantification of percent hatchlings relative to mock of treatments represented in **b** plus *E. coli* triple mutant *upp;udk;udp*. n=3. (**d**) Quantification of % hatchlings of *C. elegans* exposed from L1 to Lth-FUdR (1μg/mL) in the presence of the following metabolites: Uracil 2.5mg/mL, Uridine 5mg/mL, UMP 2.5mg/mL, UDP 5mg/mL, or UTP 5mg/mL. n=3. (**e**) Quantification of total number of embryos (all dead) laid in 24h per worm treated with Lth-FUdR ± the Tmk inhibitor 5'-iodo-UMP (100μM). n=3. (**f**) Quantification of % hatchlings in worms cultured on WT (BW25113), *upp;udk* double KO, or *upp;udk;udp* triple KO lawns and treated with increasing doses of FUdR (0-40 μg/mL). FUdR dose is depicted in logarithmic scale.

Supplemental Figure 2

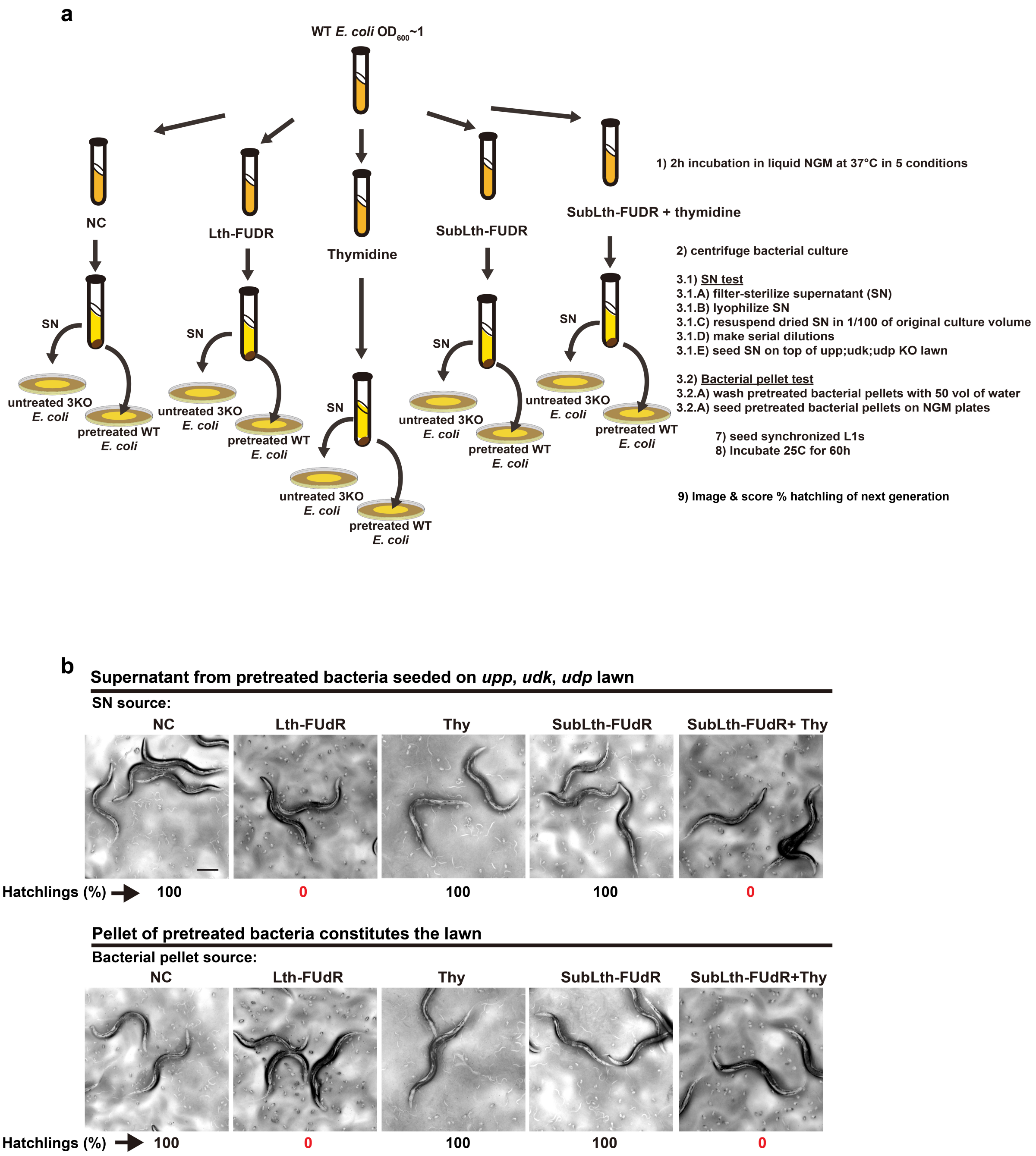

Supplementary Figure 2. Pretreating *E. coli* with thymidine is sufficient to increase FUdR toxicity in *C. elegans*.

(**a**) Workflow of "in tube" pretreatment of *E. coli* with mock, Lth-FUdR, or subLth-FUdR ± 5mg/mL thymidine and the follow up secreted versus intracellular (pellet) toxicity test. (**b**) Representative images of effect of pretreated supernatants and bacterial pellets on *C. elegans* progeny viability. Scale bars = 200μm. n=3.

Supplemental figure 3

a (copy of a portion of Suppl. Fig. 1a to avoid moving back and forth between figures)

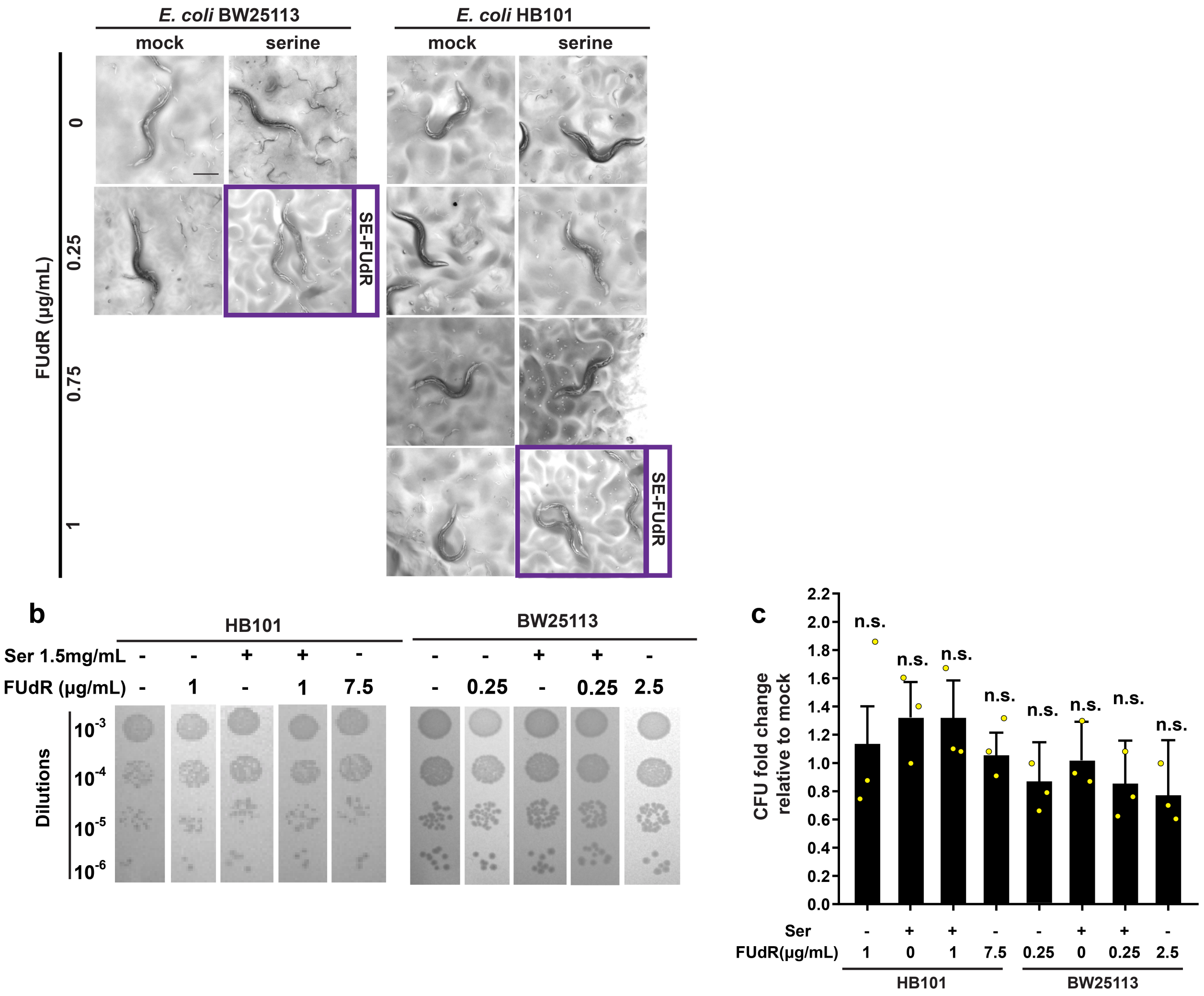

Supplementary Figure 3. Lth-FUdR and SE-FUdR toxicity do not correlate with *E. coli* in plate growth or viability.

Data acquisition and analysis described in Methods. Statistical significance for ratio CFU was assessed using one-tail ratio t-test. Data are presented as mean values ± SEM. n= # independent biological replicates. Source data are provided as a Source Data file.

(**a**) Images from Supplementary Fig. 1a are reproduced here to avoid moving back and forth between supplementary figures. This is reproduced here to show what we define as SE-FUdR toxicity, which corresponds to 1μg/mL FUdR + 1.5mg/mL serine for worms cultured on BW25113 or its derivatives, and 7.5μg/mL FUdR + 1.5mg/mL serine for worms cultured on HB101 or its derivatives (i.e. EORB1). Scale bars = 200μm. n>10. (**b**) Representative images of viability of *E. coli* BW25113 and the HB101-derivative EORB1 after incubation for 48h in NGM plates supplemented with mock, subLth-FUdR, serine, subLth-FUdR plus serine, or Lth-FUdR as assessed by serial dilution for counting of colony forming units (CFU). (**c**) Quantification of CFU of treatments represented in panel **b**. Data were analyzed using ratio t-test. n=3.

Supplemental Figure 4

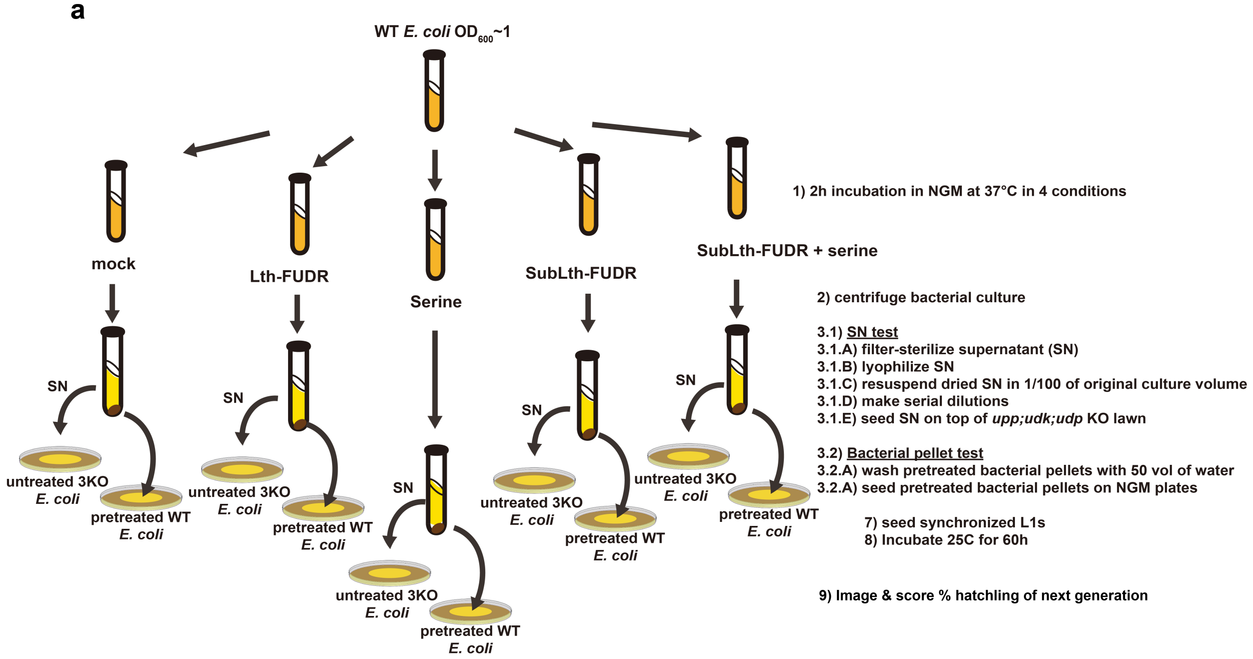

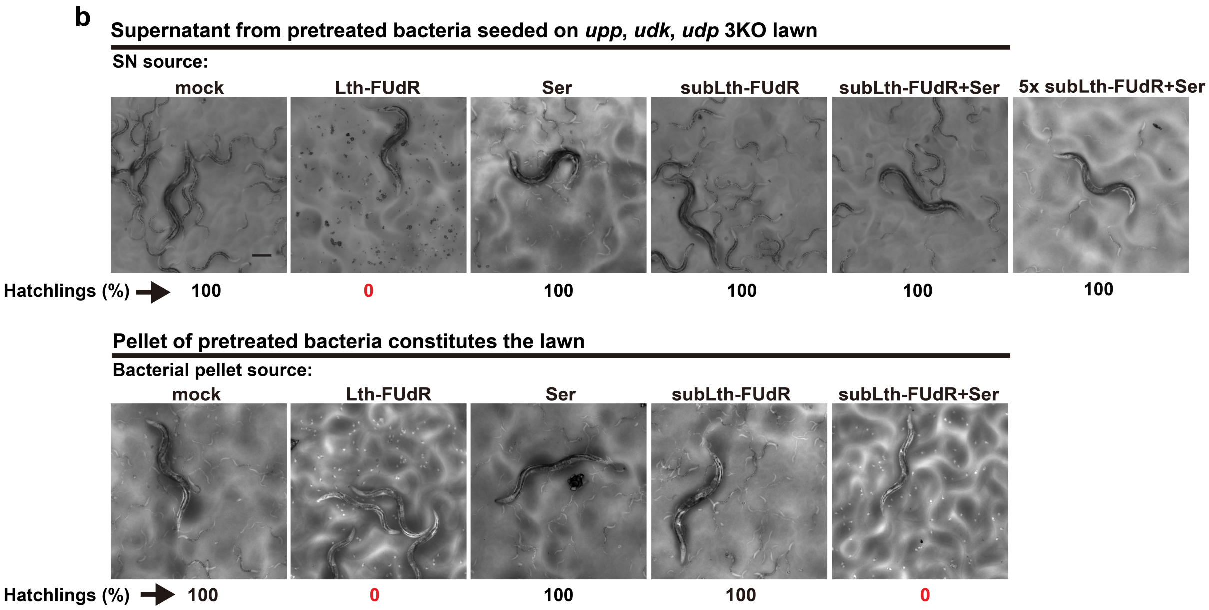

Supplementary Figure 4. Treating Pretreating *E. coli* with serine is sufficient to increase FUdR toxicity in *C. elegans*.

(**a**) Workflow of "in tube" pretreatment of *E. coli* with mock, Lth-FUdR, or subLth-FUdR ± serine and the follow up secreted versus intracellular (pellet) toxicity test. (**b**) Representative images of effect of pretreated supernatants and bacterial pellets on *C. elegans* progeny viability. Scale bars = 200μm. n=3.

Supplemental Figure 5

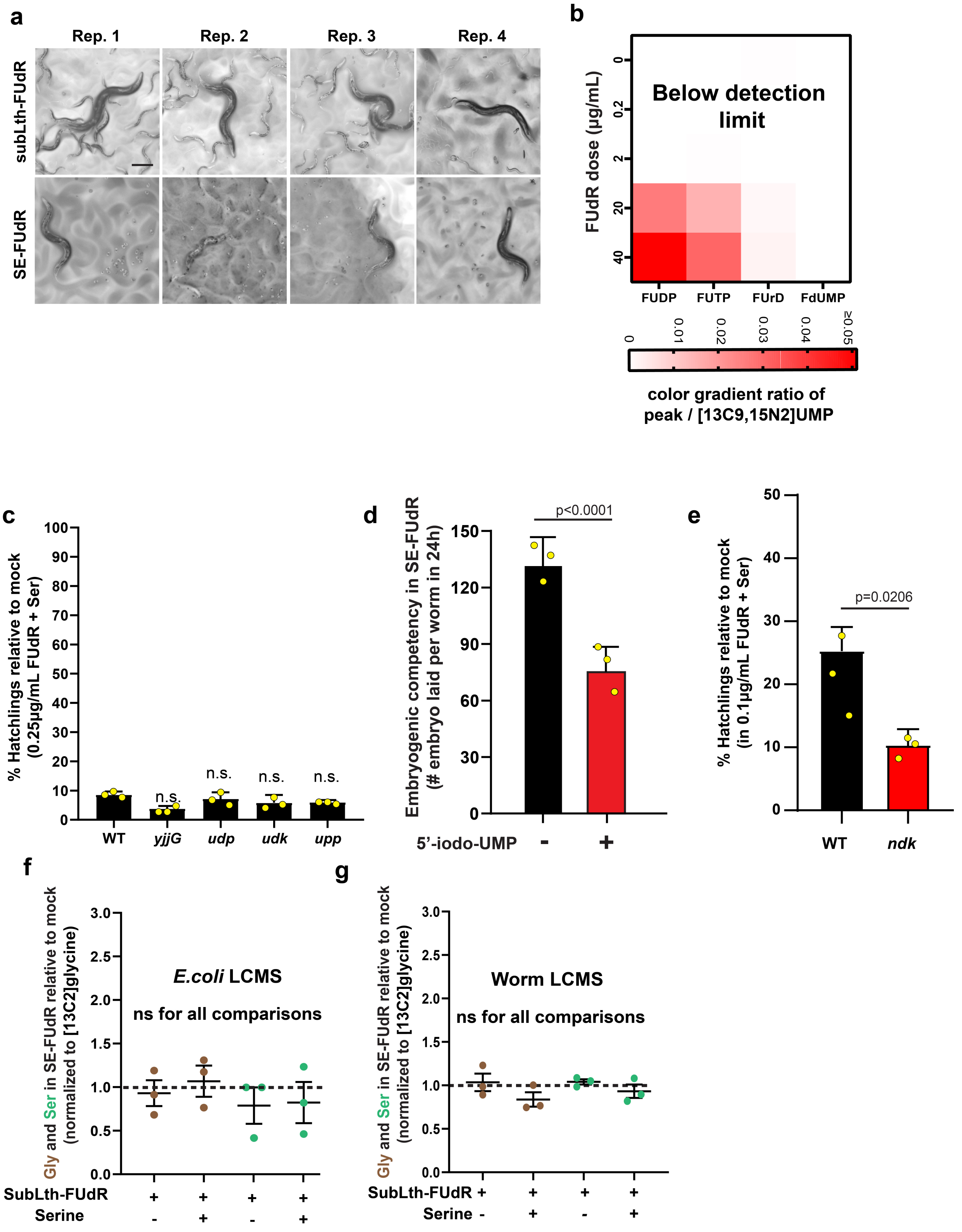

Supplementary Figure 5. Serine does not promote FUdR-to-FUMP conversion or accumulates in *E. coli.*

Throughout this figure: % hatchlings is estimated and data analyzed as described in Supplementary Fig. 1. Statistical significance was assessed via two-tailed unpaired nonparametric t-test for % hatchlings quantification. LCMS data was analyzed using one-tailed ratio t-test after ROUT outlier treatment. Data are presented as mean values ± SEM, scale bars = 200µm. n= # independent biological replicates. Source data are provided as a Source Data file.

(**a**) Representative images of progeny viability observed in worms cultured on aliquots of *E. coli* pellets used for LCMS analyses presented in Fig. 2g,h, 4g, and Supplementary Fig. 5f,g. (**b**) Representation of the capacity of our LCMS set up to detect FUDP, FUTP, FUrD and FdUMP in extracts *E. coli* (BW25113) treated with increasing doses of FUdR (0-40µg/mL). FUDP and FUTP cannot be reliably detected in samples treated with less than 20µg/mL FUdR. FUrD and FdUMP cannot be reliably measured even in samples treated with 40µg/mL FUdR (>150 fold higher than the dose we use to characterize SE-FUdR toxicity) (**c**) Quantification of % hatchlings relative to mock of *C. elegans* cultured on WT (BW25113), *yjjG*, *udp*, *udk*, or *upp* KO lawns treated from L1 with mock or 0.25µg/mL FUdR plus serine. n=3. (**d**) Quantification of total number of embryos (all dead) laid in 24h per worm treated with SE-FUdR ± the Tmk inhibitor 5'-iodo-UMP (100µM). n=3. Data were analyzed using two-tailed unpaired nonparametric T-test. (**e**) Quantification of % hatchlings relative to mock of *C. elegans* cultured on WT (BW25113) or *ndk* KO lawns treated from L1 with mock or 0.1µg/mL FUdR plus serine (lower dose of FUdR used to enable detection of toxicity enhancer). n=3. (**f-g**) LC-MS measurement of serine (green circles) and glycine (brown circles) integrated peaks normalized to internal standard [13C2] glycine in (**f**) *E. coli* (WT BW25113) and (**g**) *C. elegans* cultured on WT *E. coli* BW25113, and treated with subLth-FUdR + 1.5 mg/mL serine relative to mock, n=3. Data were analyzed using ratio t-test.

Supplemental Figure 6

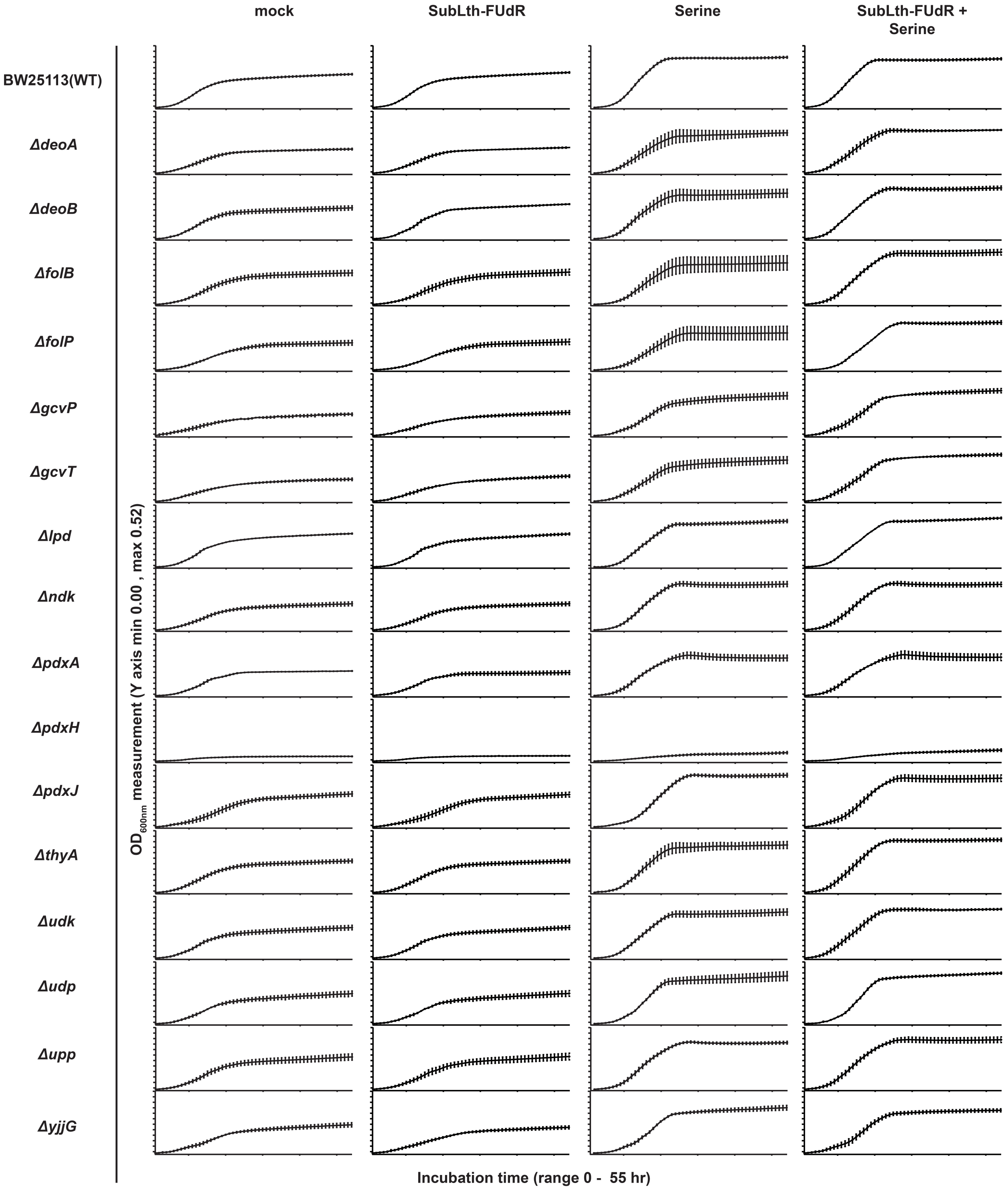

Supplementary Figure 6. *E. coli*'s capacity to suppress SE-FUdR toxicity in *C. elegans* does not correlate with growth.

Throughout this figure, data are presented as mean values ± SEM. Curves depict 55 h continuous biomass measurement (OD_{600nm}) of *E. coli* WT (BW25113) and KOs that alter SE-FUdR toxicity. Bacteria were cultured in complete liquid NGM containing mock, subLth-FUdR, serine, or subLth-FUdR plus serine. n=5. Source data are provided as a Source Data file.

Supplemental Figure 7

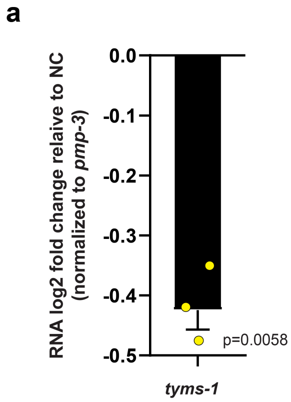

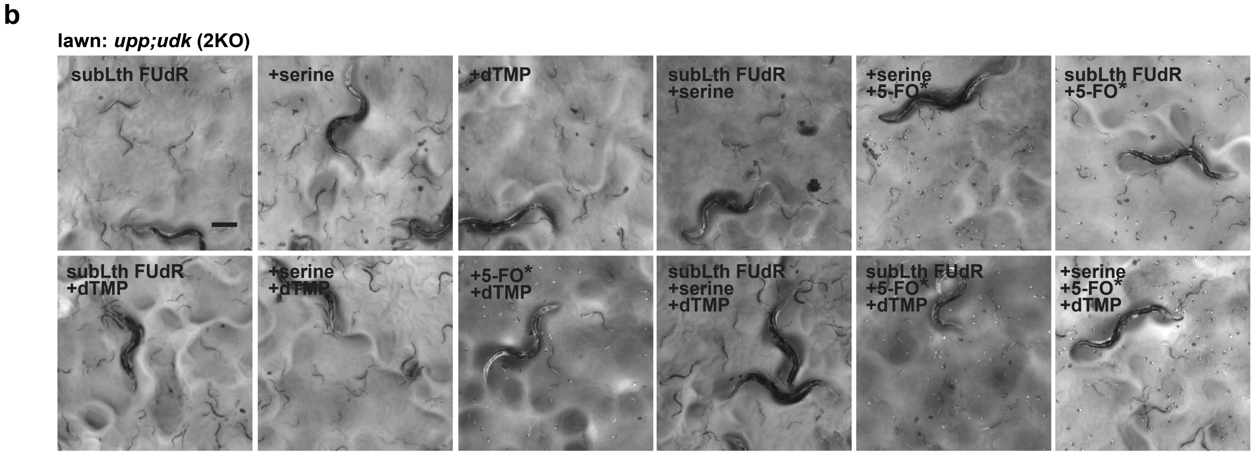

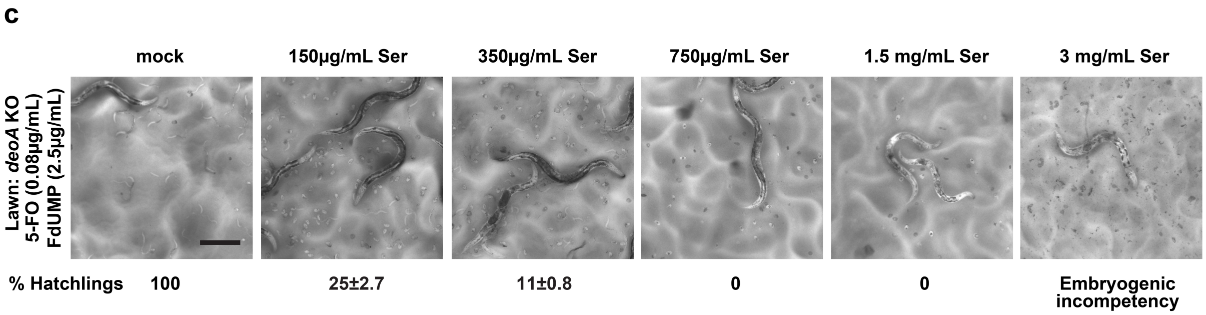

Supplementary Figure 7. Related to Fig. 4; Serine availability limits FUdR capacity to inhibit *E. coli* ThyA and hence to reduce dTMP pool in *C. elegans* diet.

Throughout this figure: data are presented as mean values ± SEM, scale bars = 200µm. n= # independent biological replicates. Source data are provided as a Source Data file.

(**a**). qRT-PCR analysis of aliquots of *tyms-1* RNAi and EORB1 EV worms used in Fig 4a. Fold change of *tyms-1* expression was calculated as ΔΔCT as described in Methods, using *pmp-3* as reference gene. Data were analyzed using one-tailed ratio t-test, and are presented as log2 of fold change. n=3. (**b**) Representative images of all control conditions in Fig. 4j. n=3. (**c**) Representative images of effect of increasing doses of serine (0-3mg/mL) on *C. elegans* exposed to 0.08µg/mL 5-FO (to provide FUMP through a *upp*,*udk*-independent pathway) and 2.5µg/mL FdUMP (the main mediator of thymidine-less death) while cultured on *deoA* KO lawn (to avoid conversion of FdUMP into other fluoropyrimidines). n=3.

Supplemental Figure 8

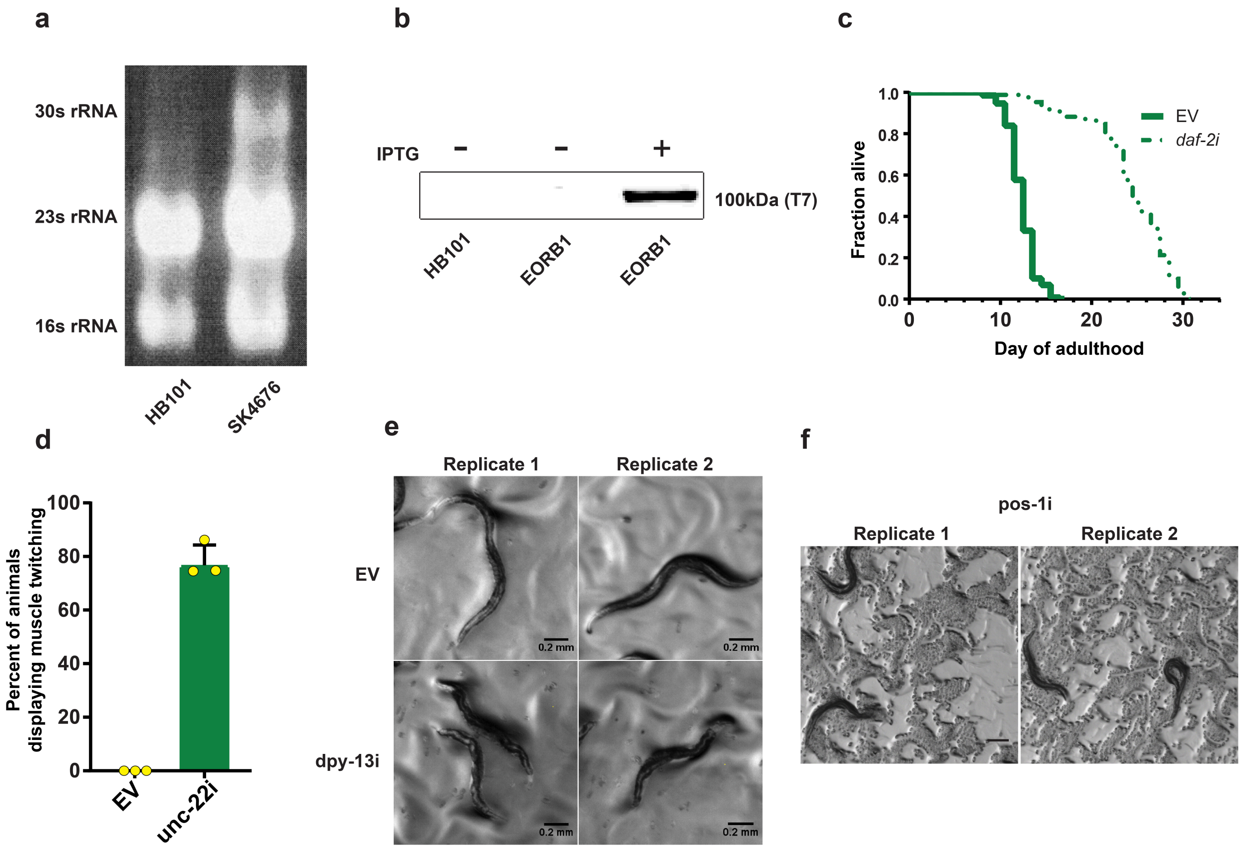

Supplementary Figure 8. EORB1 is an RNAi-competent derivative of *E. coli* HB101.

(**a**) HB101 *rnc*- derivative, but not HB101 parental, accumulates 30S rRNA precursor, n = 1. (**b**) IPTG-inducible T7 polymerase is expressed in EORB1 but not in the HB101 parental strain, n = 1. (**c-f**) EORB1 is competent for feeding RNAi: (**c**) RNAi against *daf-2* extends *C. elegans* lifespan ~2-fold, n = 2, (**d**) *unc-22* causes muscle twitching in >75% of animals. n=3, data is presented the as mean value ± SEM. (**e**) *dpy-13* causes a dumpy phenotype in 100% of animals (2 representative experiments shown for control and RNAi), scale bars = 200μm, and (**f**) *pos-1* causes 100% sterility (2 representative experiments shown for RNAi) scale bars = 200μm. Source data are provided as a Source Data file.

Supplemental Figure 9

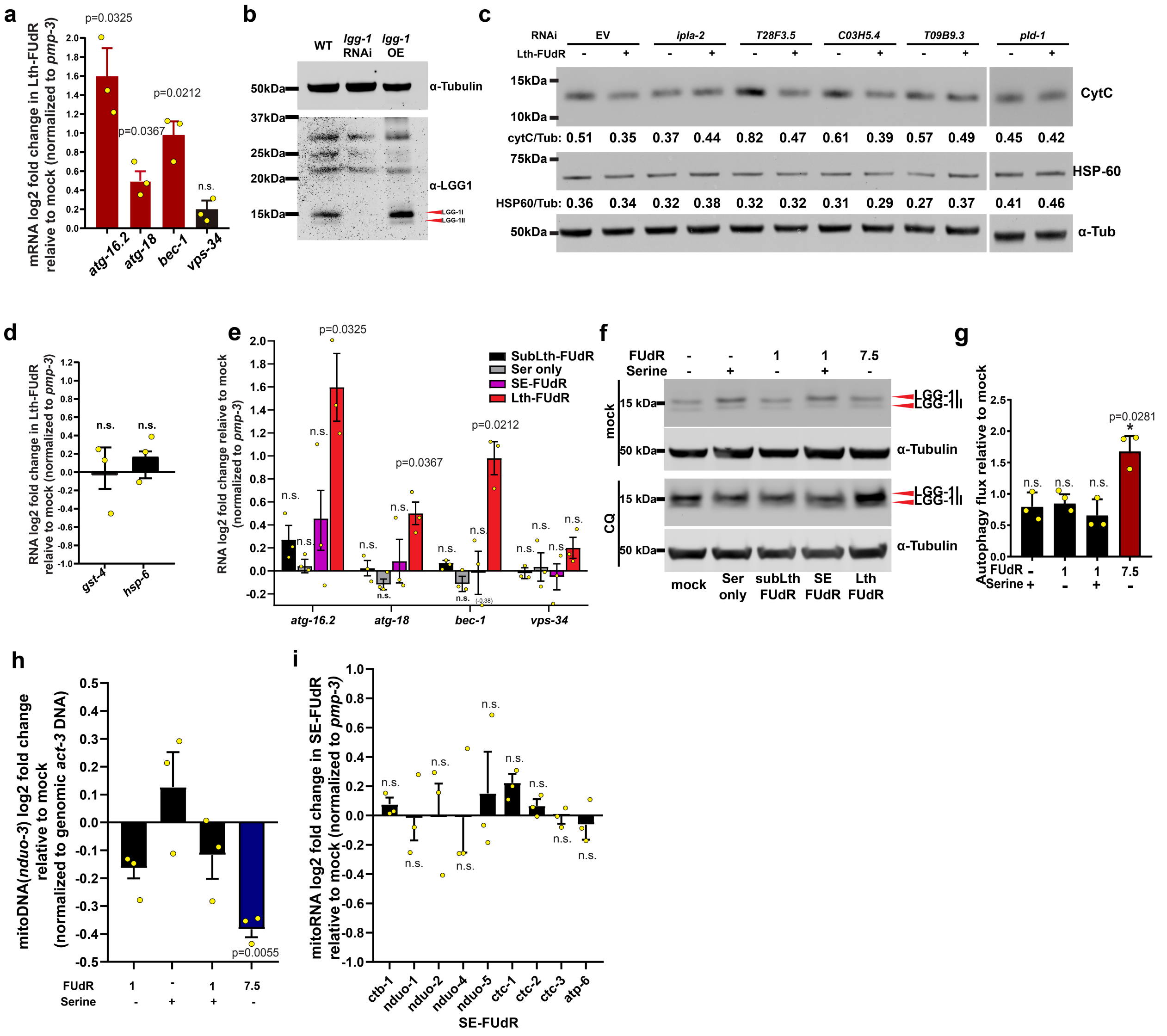

Supplementary Figure 9. Related to Fig. 7; SE-FUdR does not activate autophagy or alters mitochondrial function

Throughout this figure: statistical significance was assessed using one-tailed ratio t-test. Data are presented as mean values ± SEM. n = # independent biological replicates. Source data are provided as a Source Data file.

(**a**) qRT-PCR analysis of autophagy genes in worms cultured on EORB1 lawn and treated with Lth-FUdR. Fold change was calculated as ΔΔCT as described in Methods, using *pmp-3* as reference gene. Data are presented as log2 of fold change. n=3 (**b**) The specificity of α-LGG1 antibody was validated by western blotting of whole lysates of *C. elegans* fed RNAi against *lgg-1* and of a transgenic line overexpressing *lgg-1* (MAH215). α-Tubulin (4A1) was used as loading reference. Red arrows point to LGG1I and LGG1II bands. n=1 (**c**) Representative western blotting analysis of endogenous cytochrome C (cytC) and of HSP-60 in worms treated with Lth-FUdR and fed EORB1 EV or RNAi targeting mitochondrial genes that suppress Lth-FUdR toxicity. Quantification of band intensity relative to α-Tubulin is depicted. n=3. (**d**) qRT-PCR analysis of mito oxidative stress response (*gst-4*) and mitoUPR (*hsp*-6) in worms treated with Lth-FUdR relative to mock. Analysis and quantification as described in panel **a**. n=3. (**e**) qRT-PCR analysis of autophagy genes in worms cultured on EORB1 lawn and treated with subLth-FUdR, serine, SE-FUdR, or Lth-FUdR. Analysis and quantification as described in panel **a**. n=3. (**f**) Western blotting analysis of α-LGG1 in worms cultured on EORB1 and treated with mock, serine, subLth-FUdR, SE-FUdR, and Lth-FUdR ± 20mM Chloroquine. (**g**) Quantification of LGG-1 relative to α-Tubulin in treatments represented in panel **f**. Analysis and quantification as described in Fig. 5h n=3. Data analyzed using ratio t-test. (**h**) qPCR analysis of mitochondrial DNA content (*nduo-3*) relative to nuclear DNA (*act-3*) in subLth-FUdR, serine, SE-FUdR and Lth-FUdR relative to mock. Data presented as log2 fold change and statistically analyzed using ratio t-test. n=3. (**i**) qRT-PCR analysis of the expression/stability of mitochondrially-encoded genes in worms treated with SE-FUdR. Quantification and analysis as described in panel **a**. n=3.

Supplemental Figure 10

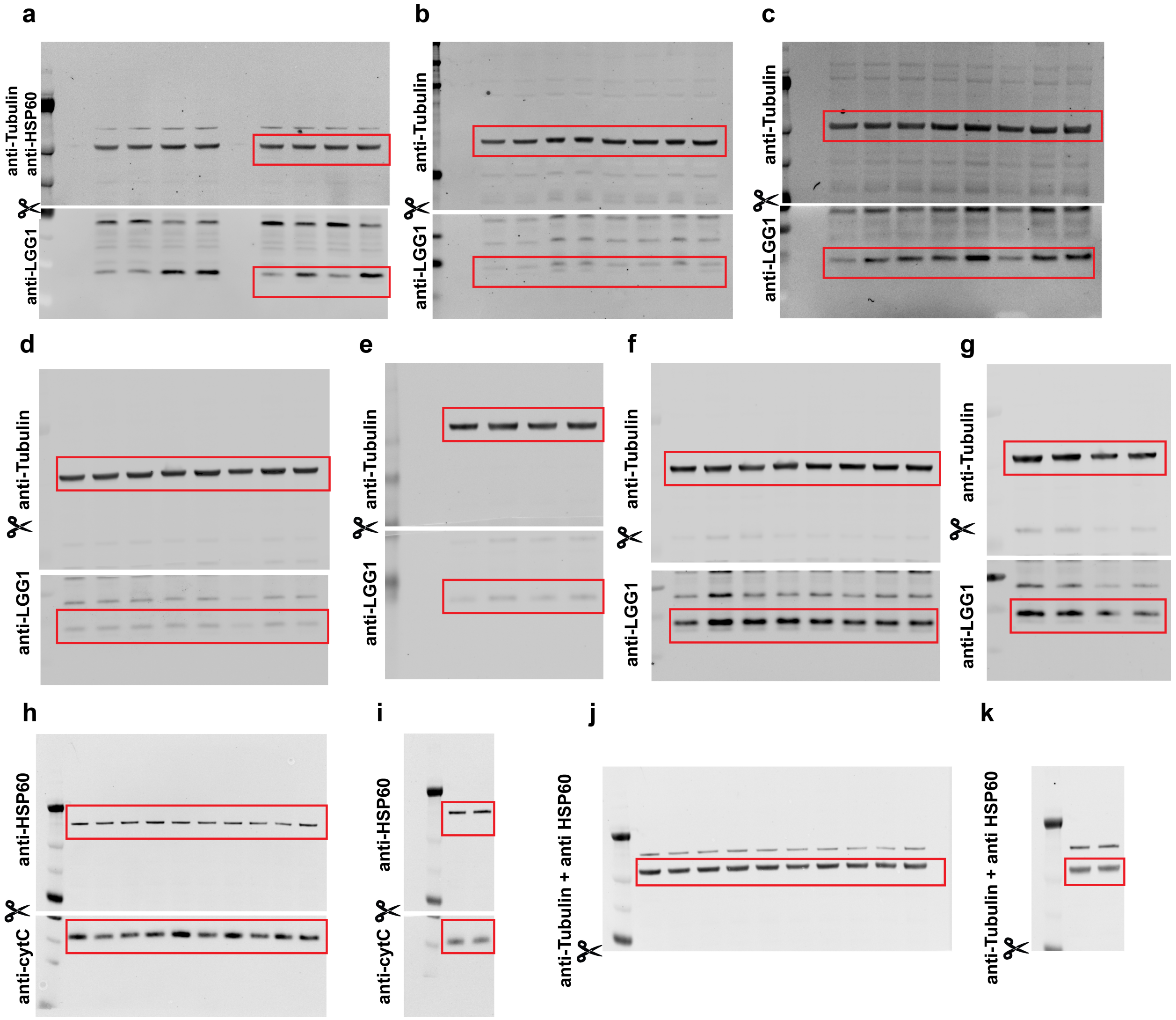

Supplementary Figure 10. Full western blots presented in main figures.

(**a**) Full blot of Fig. 5d. (**b**, **c**) Full blots of Fig. 5h (**d-g**) Full blots of Fig. 6c. (**h-k**) Full blots of Supplementary Figure 9c. Red boxes denote section cropped for display.

Supplementary Table 1

Gene name	B number	Gene name	B number	Gene name	B number
alsA	b4087	*mrcB*	b0149	*sdaA*	b1814
alsB	b4088	*mutM*	b3635	*sdaB*	b2797
alsC	b4086	*nadC*	b0109	*sdaC*	b2796
aphA	b4055	*ndk*	b2518	*serA*	b2913
apt	b0469	*nei*	b0714	*serB*	b4388
cdd	b2143	*nlpD*	b2742	*serC*	b0907
cmk	b0910	*nrdD*	b4238	*solA*	b1059
codA	b0337	*nth*	b1633	*sstT*	b3089
codB	b0336	*nupC*	b2393	*surE*	b2744
cpdA	b3032	*nupG*	b2964	*tdcC*	b3116
cpdB	b4213	*ompC*	b2215	*tdcG*	b4471
crp	b3357	*ompF*	b0929	*tdk*	b1238
cyaA	b3806	*ompN*	b1377	*thiD*	b2103
cycA	b4208	*pabA*	b3360	*thiE*	b3993
cysE	b3607	*pabB*	b1812	*thyA*	b2827
damX	b3388	*pabC*	b1096	*tnaA*	b3708
dcd	b2065	*pdxA*	b0052	*trpA*	b1260
dedD	b2314	*pdxH*	b1638	*trpB*	b1261
deoA	b4382	*pdxJ*	b2564	*trpD*	b1263
deoB	b4383	*pdxK*	b2418	*tsx*	b0411
deoD	b4384	*pdxY*	b1636	*udk*	b2066
dksA	b0145	*pepA*	b4260	*udp*	b3831
dosP	b1489	*pepB*	b2523	*ugpQ*	b3449
entF	b0586	*pepD*	b0237	*upp*	b2498
fis	b3261	*pepN*	b0932	*uraA*	b2497
folB	b3058	*phnN*	b4094	*ushA*	b0480
folM	b1606	*phoB*	b0399	*xapA*	b2407
folP	b3177	*phoE*	b0241	*xapB*	b2406
fruR	b0080	*pncB*	b0931	*yaaJ*	b0007
gcvH	b2904	*prkB*	b3355	*ybiV*	b0822
gcvP	b2903	*purD*	b4005	*ybjI*	b0844
gcvT	b2905	*purF*	b2312	*yccR*	b0959
glpQ	b2239	*purH*	b4006	*ycdG*	b1006
glyA	b2551	*purN*	b2500	*ydfG*	b1539
glyS	b3559	*purR*	b1658	*yeaV*	b1801
gpt	b0238	*purT*	b1849	*yeiA*	b2147
gshB	b2947	*purU*	b1232	*yeiT*	b2146
hisG	b2019	*pyrE*	b3642	*yfaO*	b2251
hpt	b0125	*pyrF*	b1281	*yfbR*	b2291
ihfA	b1712	*rbsA*	b3749	*ygfA*	b2912
ihfB	b0912	*rbsB*	b3751	*yggC*	b2928
kbl	b3617	*rbsC*	b3750	*ygjF*	b3068
lpd	b0116	*rbsD*	b3748	*yhfW*	b3380
lpp	b1677	*rbsK*	b3752	*yigB*	b3812
ltaE	b0870	*recX*	b2698	*yjfF*	b4231
mazG	b2781	*rfe*	b3784	*yjjG*	b4374
metF	b3941	*rihA*	b0651	*ytfQ*	b4227
metH	b4019	*rihB*	b2162	*ytfR*	b4485
mrcA	b3396	*rihC*	b0030	*ytfT*	b4230

Supplementary Table 1. List of *E. coli* genes screened from the Keio *E. coli* knock-out library. List is comprised of genes found by iJO1366 model to be associated 1-2 steps away from homologs of mammalian fluoropyrimidine metabolic pathways. In total 147 loss of function mutants in the background *E. coli* strain BW25113 were screened.

Supplementary Table 2

Gene name	Keio clone	Biological process (EC)	Verified
codA	b0337	Deamination of cytosine to uracil (3.5.4.1)	NRT
crp	b3357	cAMP receptor protein, regulates transcription	√
cysE	b3607	Serine synthesis (2.3.1.30)	NRT
dcd	b2065	dUTP synthesis from dCTP (3.5.4.13)	NRT
deoA	b4382	thymine/uracil synthesis from thymidine/deoxyuridine (2.4.2.4)	√
deoB	b4383	PRPP synthesis (5.4.2.7)	NRT
dksA	b0145	Regulates rRNA transcription	NRT
dosP	b1489	c-di-GMP hydrolysis (3.1.4.52)	NRT
folB	b3058	THF synthesis (4.1.2.25)	√
folP	b3177	THF synthesis (2.5.1.15)	√
gcvP	b2903	10fTHF synthesis (1.4.4.2)	√
gcvT	b2905	10fTHF synthesis (2.1.2.10)	√
lpd	b0116	10fTHF synthesis (1.8.1.4)	√
metF	b3941	5,10-methylene-THF metabolism (1.5.1.20)	X
nlpD	b2742	Divisome associated factor	NRT
pdxA	b0052	Pyridoxal-5-phosphate synthesis	√
pdxH	b1638	Pyridoxal-5-phosphate synthesis and salvage (1.4.3.5)	√
pdxJ	b2564	Pyridoxal-5-phosphate synthesis (2.6.99.2)	√
pepD	b0237	Muropeptide degradation (3.4.13.18)	NRT
phoE	b0241	Outer membrane phosphate transport	NRT
rbsK	b3752	Ribose degradation (2.7.1.15)	NRT
serC	b0907	Serine synthesis (2.6.1.52)	√
upp	b2498	UMP salvage 2.4.2.9	X
yjjG	b4374	Pyrimidine nucleotidase (3.1.3.5)	X

Gene name	Keio clone	Biological process (EC)	Verified
ndk	b2518	UTP/CTP synthesis (2.7.4.6)	√
pabC	b1096	PABA/tetrahydrofolate syNRThesis (4.1.3.38)	NRT
sdaB	b2797	Serine degradation	NRT
**udk**	b2066	UMP/CMP salvage (2.7.1.48)	X
**udp**	b3831	Uracil salvage (2.4.2.3)	X

Supplementary Table 2. Hits of Keio screen for mediators of SE-FUdR toxicity.

Primary hits are presented as: blue = suppressor of toxicity; orange= enhancer of toxicity; and white= no different from WT control. Light blue or orange, represents phenotype observed in only 1-2 of 3 screen repeats. Hits belonging to overrepresented metabolic pathways were retested in 6cm NGM plates and quantitated for % hatchlings in sublethal FUdR (unless otherwise stated 0.25μg/mL) ± serine (1.5mg/mL), and the results are presented in main figures. Primary screen hits that were not retested in 6cm plates are depicted as NRT. Retested and verified hits (colored cells) and non-hits (white cells) are marked as "√", and retested but not validated primary hits (phenotype did not repeat) are marked as "X".

Supplementary Table 3

Symbol	Biological process	Lth-FUdR	SE-FUdR
vector control			
F19G12.2	DNA damage response	NRT	NRT
msh-6	mismatch repair of DNA (previously shown in Sengupta et al., 2013)	√	√
ung-1	base-excision repair of DNA	√	√
pus-1	Catalyzes pseudouridylation. Toxic covalent complex with fluorouracil.	√	√
cdd-1	cytidine/deoxycytidine conversion to uridine/deoxyuridine	NRT	NRT
cdd-2	cytidine/deoxycytidine conversion to uridine/deoxyuridine	NRT	NRT
H24K24.3	alcohol dehydrogenase providing formate for pyrimidine synthesis	NRT	NRT
K02D7.1	purine nucleoside phosphorylase	NRT	NRT
tyms-1	thymidine synthesis	X	X
aak-2	AMPK subunit	√	√
atg-7	Autophagy (previously shown in Sengupta et al., 2013)	X	X
bec-1	Autophagy (previously shown in Sengupta et al., 2013)	√	√
lgg-1	Autophagy	√	√
lgg-2	Autophagy	√	√
vps-34	Autophagy	√	√
T28F3.5	conversion of acetyl-CoA to malonyl-CoA (lipogenesis)	√	√
ipla-2	similar to phospholipase A2	√	√
C03H5.4	similar to phospholipase A2	√	√
pld-1	similar to phospholipase D1	√	√
T09B9.3	glycerophosphodiesterase	√	√
aat-2	amino acid transport	NRT	NRT
aat-3	amino acid transport	NRT	NRT
C15B12.1	lysine catabolism	NRT	NRT
cysl-1	cysteine synthesis	NRT	NRT
cysl-4	cysteine synthesis	NRT	NRT
got-2.2	aspartate catabolism	NRT	NRT
pstk-1	seryl-tRNA phosphorylation	NRT	NRT
snf-6	amino acid transport	NRT	NRT
fmo-2	Detox response	NRT	NRT
daf-16	Detox transcription factor	NRT	NRT
C49F5.5	histone acetylation	NRT	NRT
pcs-1	Detox response	NRT	NRT
skn-1	Detox transcription factor	NRT	NRT
mpst-1	iron-sulfur complex formation	NRT	NRT
Y45F10D.4	iron homeostasis	NRT	NRT
ctl-2	oxidative stress response	NRT	NRT
gst-3	oxidative stress response	NRT	NRT
gpx-8	oxidative stress response	NRT	NRT
gpx-5	oxidative stress response	NRT	NRT

Supplementary Table 3. *C. elegans* suppressors and enhancers of Lth-FUdR and SE-FUdR toxicity

Primary RNAi hits are presented as: blue = suppressor of toxicity; orange= enhancer of toxicity; and white= no different from WT control. Light blue or orange, represents phenotype observed in only 1-2 of 3 screen repeats. Hits belonging to overrepresented metabolic pathways were retested in 6cm NGM plates and quantitated for % hatchlings in sublethal FUdR (unless otherwise stated 1μg/mL) ± serine (1.5mg/mL), and the results are presented in main figures. Primary screen hits that were not retested in 6cm plates are depicted as NRT. Retested and verified hits (colored cells) and non-hits (white cells) are marked as "√", and retested but not validated primary hits (phenotype did not repeat) are marked as "X".

Supplementary Note 1

Since the SE-FUdR mechanism of toxicity seems distinct from Lth-FUdR and TE-FUdR, fluororibonucleotides other than FUMP, specifically FUDP, FUTP, or fluorouridine (FUrd), could contribute to SE-FUdR toxicity. However, these fluororibonucleotides were below the detection limit of our LCMS of bacteria or worms treated with subLth-FUdR or SE-FUdR (Supplementary Fig. 5b).

This is not surprising, as previous studies[1] used a dose of fluoropyrimidine 50 fold higher (50µM) than the 0.25µg/mL (1µM) FUdR we use to study SE-FUdR toxicity.

SE-FUdR toxicity cannot be biochemically assessed at higher fluoropyrimidine doses because:

1) When using *E. coli* BW25113, doses of FUdR ≥1µg/mL lead to 100% sterility. Hence, serine enhancement of toxicity cannot be assessed because further enhancement of sterility cannot be achieved; and

2) Although serine-enhanced toxicity can be observed at >10µg/mL FUdR in the form of developmental delay, it would not be informative to compare the metabolic profile of adults to young larvae.

Nevertheless, we used genetic and chemical inhibitor approaches to assess the potential contribution of other fluororibonucleotides, including FUDP, FUTP, FUrd, and FdUMP to SE-FUdR toxicity, and directly measured the levels of FUMP and 5-FU.

Supplementary Note 2

A lower dose of subLth-FUdR was used in this experiment to enable detection of enhancement of toxicity when combining serine with *thyA*. The results also suggest that *C. elegans* is more dependent on *E. coli*'s supply of dTMP than its own *de novo* synthesis pathway, and that inhibition of *E. coli*'s TS is not detrimental to worms unless another concurrent insult is present (e.g. sublethal levels of FUMP toxicity).

Supplementary Note 3

Set up of experiment aimed to test whether SE-FUdR toxicity can be rescued by dietary supplementation of dTMP.

This test requires a complex experimental set up because dTMP enhances FUdR-to-FUMP conversion, and hence, toxicity in *C. elegans*. So, if we simply add FUdR + serine + dTMP to w

wild-type *E coli* lawn we will see dTMP enhances, instead of rescuing the toxicity. But this would be due to confounding factors.

Hence, to test whether SE-FUdR toxicity can be rescued by dietary supplementation of dTMP we need to prevent FUdR-to-FUMP conversion. This can be easily achieved by using a *upp,udk* double KO or a *upp,udk,udp* triple KO *E. coli* lawn. However, this brings another challenge. That is that we need sublethal levels of FUMP toxicity for SE-FUdR toxicity to work; hence, if using *E coli* lawns unable to convert FUdR into FUMP, we need an alternative source of FUMP. The simplest would be to add FUMP to the system. However, FUMP is commercially available only as custom–made at the microgram level and it is not affordable. To overcome the need to provide FUMP in the absence of the FUdR-to-FUMP conversion pathway, we use 5'-fluorotic acid (5-FO) as the source of FUMP, as *E. coli* converts 5-FO into FUMP in a *upp;udk* independent-manner [1].

Therefore, altogether the experimental set up to test whether SE-FUdR toxicity can be rescued by dietary supplementation of dTMP includes:

1) *upp;udk* double KO lawn to avoid dTMP + FUdR promoting FUMP toxicity;

2) 5'-fluorotic acid (5-FO) as the source of FUMP that is independent from Upp/Udp-Udk

3) SubLth-FUdR + serine to promote SE-FUdR; and

4) dTMP (1.5μg/mL) or mock to test whether dTMP supplementation rescues of SE-FUdR toxicity.

This complex experimental setup is necessary to test whether dietary thymidine rescues SE-FUdR because otherwise thymidine would simultaneously enhance Lth-FUdR toxicity (Fig. 1g-n), yielding confounding results.

Supplementary References

1. Scott, T. A. *et al.* Host-Microbe Co-metabolism Dictates Cancer Drug Efficacy in *C. elegans*. *Cell* **169**, 442-456.e18 (2017).
