# Supplementary material for: Dietary serine-microbiota interaction enhances chemotherapeutic toxicity without altering drug conversion
Source: Nat Commun. 2020 May 22;11:2587. doi: 10.1038/s41467-020-16220-w (PMC7244588; doi:10.1038/s41467-020-16220-w)
Supplement: Supplementary file 2 — Description of Additional Supplementary Files [file 41467_2020_16220_MOESM2_ESM.pdf]

Description of Additional Supplementary Files

File Name: Supplementary Data 1

Description: List of C. elegans genes tested in the EORB1 Lth-FUdR, and SE-FUdR RNAi screens. 387 gene knockdowns were tested in duplicate or triplicate in Lth-FUdR and SE-FUdR.
