# Supplementary material for: Dietary serine-microbiota interaction enhances chemotherapeutic toxicity without altering drug conversion
Source: Nat Commun. 2020 May 22;11:2587. doi: 10.1038/s41467-020-16220-w (PMC7244588; doi:10.1038/s41467-020-16220-w)
Supplement: Supplementary file 4 — Reporting Summary [file 41467_2020_16220_MOESM4_ESM.pdf]

nature research

Corresponding author(s): Eyleen O'Rourke

Last updated by author(s): Mar 28, 2020

Reporting Summary

Nature Research wishes to improve the reproducibility of the work that we publish. This form provides structure for consistency and transparency in reporting. For further information on Nature Research policies, see Authors & Referees and the Editorial Policy Checklist .

Statistics

For all statistical analyses, confirm that the following items are present in the figure legend, table legend, main text, or Methods section.

n/a	Confirmed	
☐	☒	The exact sample size (*n*) for each experimental group/condition, given as a discrete number and unit of measurement
☐	☒	A statement on whether measurements were taken from distinct samples or whether the same sample was measured repeatedly
☐	☒	The statistical test(s) used AND whether they are one- or two-sided<br>*Only common tests should be described solely by name; describe more complex techniques in the Methods section.*
☐	☒	A description of all covariates tested
☒	☐	A description of any assumptions or corrections, such as tests of normality and adjustment for multiple comparisons
☐	☒	A full description of the statistical parameters including central tendency (e.g. means) or other basic estimates (e.g. regression coefficient) AND variation (e.g. standard deviation) or associated estimates of uncertainty (e.g. confidence intervals)
☐	☒	For null hypothesis testing, the test statistic (e.g. *F*, *t*, *r*) with confidence intervals, effect sizes, degrees of freedom and *P* value noted<br>*Give P values as exact values whenever suitable.*
☒	☐	For Bayesian analysis, information on the choice of priors and Markov chain Monte Carlo settings
☒	☐	For hierarchical and complex designs, identification of the appropriate level for tests and full reporting of outcomes
☒	☐	Estimates of effect sizes (e.g. Cohen's *d*, Pearson's *r*), indicating how they were calculated

Our web collection on statistics for biologists contains articles on many of the points above.

Software and code

Policy information about availability of computer code

Data collection	We do not use computer coding in this study
Data analysis	We do not use computer coding in this study

For manuscripts utilizing custom algorithms or software that are central to the research but not yet described in published literature, software must be made available to editors/reviewers. We strongly encourage code deposition in a community repository (e.g. GitHub). See the Nature Research guidelines for submitting code & software for further information.

Data

Policy information about availability of data

All manuscripts must include a data availability statement. This statement should provide the following information, where applicable:

- Accession codes, unique identifiers, or web links for publicly available datasets
- A list of figures that have associated raw data
- A description of any restrictions on data availability

The data that support the findings of this study are available from the corresponding author upon request. Figures that have associated Raw data are: 1b-m, 2b-j, 3b-c, 4a-g, 4i-j, 5a-i, 6a-h, 7a-g, S1b-f, S2b, S3b-c, S4b, S5a-e, S6, S7a-e, S8a-f, S9a-i, S10a-k, Table S1-S3. All reagents generated in this study and necessary to replicate the findings (i.e. EORB1 E. coli strain) are available from the corresponding author upon request.

Field-specific reporting

Please select the one below that is the best fit for your research. If you are not sure, read the appropriate sections before making your selection.

Life sciences study design

All studies must disclose on these points even when the disclosure is negative.

Sample size	We followed standards for C. elegans including (per treatment/per replicate): 1) ~500 worms to measure progeny viability, 2) ~10,000 worms for LCMS, 3) ~2,500 worms for western blotting, 4) ≥15 worms for 3D confocal reconstruction, 5) >50 worms for lifespan assays.
Data exclusions	Data were not excluded
Replication	All experiments were independently replicated at least 3 times. Independent means C. elegans cultures, E. coli streaks, culture plates, reagents, etc, were prepared fresh and separated in time for each replicate.
Randomization	E. coli and C. elegans were aliquoted and randomly assigned as controls or treatment subjects.
Blinding	Blinding was applied to the imaging and quantification of autophagy. Additionally, the images of populations of control and treated worms used for score the viability and development were quantitated by researchers that did not capture the images or knew what each image corresponded to.

Reporting for specific materials, systems and methods

We require information from authors about some types of materials, experimental systems and methods used in many studies. Here, indicate whether each material, system or method listed is relevant to your study. If you are not sure if a list item applies to your research, read the appropriate section before selecting a response.

Materials & experimental systems

n/a	Involved in the study	
☐	☒	Antibodies
☒	☐	Eukaryotic cell lines
☒	☐	Palaeontology
☐	☒	Animals and other organisms
☒	☐	Human research participants
☒	☐	Clinical data

Methods

n/a	Involved in the study	
☒	☐	ChIP-seq
☒	☐	Flow cytometry
☒	☐	MRI-based neuroimaging

Antibodies

Antibodies used	custom made anti-LGG-1
Validation	Validation of custom-made anti-LGG-1 antibody is included in paper

Animals and other organisms

Policy information about studies involving animals; ARRIVE guidelines recommended for reporting animal research

Laboratory animals	Only invertebrates were used in this study. C elegans used in this study were hermaphroditic.
Wild animals	No wild animals were used in this study
Field-collected samples	No field collection was used in this study
Ethics oversight	None of the species used here (E. coli and C. elegans) are subject to ethics regulation

Note that full information on the approval of the study protocol must also be provided in the manuscript.
